# Fifteen-Year Application of Manure and Chemical Fertilizers Differently Impacts Soil ARGs and Microbial Community Structure

**DOI:** 10.3389/fmicb.2020.00062

**Published:** 2020-02-06

**Authors:** Fenghua Wang, Wanxue Han, Shuaimin Chen, Wenxu Dong, Min Qiao, Chunsheng Hu, Binbin Liu

**Affiliations:** ^1^Key Laboratory of Agricultural Water Resources, Hebei Key Laboratory of Soil Ecology, Center for Agricultural Resources Research, Institute of Genetics and Developmental Biology, Chinese Academy of Sciences, Shijiazhuang, China; ^2^University of Chinese Academy of Sciences, Beijing, China; ^3^Institute of Agricultural Resource and Environment, Jilin Academy of Agricultural Sciences, Changchun, China; ^4^State Key Lab of Urban and Regional Ecology, Research Center for Eco-Environmental Sciences, Chinese Academy of Sciences, Beijing, China

**Keywords:** antibiotic resistance genes, mobile genetic elements, manure, chemical fertilizer, microbial community structure

## Abstract

Manure, which contains large amounts of antibiotics and antibiotic resistance genes (ARGs), is widely used in agricultural soils and may lead to the evolution and dispersal of ARGs in the soil environment. In the present study, soils that received manure or chemical fertilizers for 15 years were sampled on the North China Plain (NCP), which is one of the primary areas of intensive agriculture in China. High-throughput quantitative PCR and sequencing technologies were employed to assess the effects of long-term manure or chemical fertilizer application on the distribution of ARGs and microbial communities. A total of 114 unique ARGs were successfully amplified from all soil samples. Manure application markedly increased the relative abundance and detectable numbers of ARGs, with up to 0.23 copies/16S rRNA gene and 81 unique ARGs. The increased abundance of ARGs in manure-fertilized soil was mainly due to the manure increasing the abundance of indigenous soil ARGs. In contrast, chemical fertilizers only moderately affected the diversity of ARGs and had no significant effect on the relative abundance of the total ARGs. In addition, manure application increased the abundance of mobile genetic elements (MGEs), which were significantly and positively correlated with most types of ARGs, indicating that horizontal gene transfer via MGEs may play an important role in the spread of ARGs. Furthermore, the application of manure and chemical fertilizers significantly affected microbial community structure, and variation partitioning analysis showed that microbial community shifts represented the major driver shaping the antibiotic resistome. Taken together, our results provide insight into the long-term effects of manure and chemical fertilization on the dissemination of ARGs in intensive agricultural ecosystems.

## Introduction

The increasing dissemination and propagation of antibiotic resistance genes (ARGs) in various environments has aroused great concern worldwide (Zhu et al., 2013; Fahrenfeld et al., 2014) and might threaten antibiotic effectiveness and public health in the 21st century (Berendonk et al., 2015). China is the largest consumer of antibiotics in the world, with a total usage of approximately 162 kt in 2013, and half of the total antibiotics consumed in China were used to treat animal diseases and promote animal growth (Zhang et al., 2015). The overuse of antibiotics in modern livestock husbandry has been proven to be a potential key driver of the expansion of environmental ARG reservoirs (Boxall et al., 2003; Brandt et al., 2009).

Indeed, prior studies have demonstrated that manure is an important reservoir of antibiotic-resistant bacteria (ARB), ARGs and mobile genetic elements (MGEs) (Heuer et al., 2011; Su et al., 2014; Udikovic-Kolic et al., 2014; Chen Q. et al., 2016). Although manure fertilizers contain abundant nutrients and organic matter that can promote crop growth, the application of manure to farmland soils might markedly increase ARG abundance and ARB populations in soils (Heuer et al., 2011) by introducing new types of ARGs or elevating existing ARG levels (Udikovic-Kolic et al., 2014). Importantly, horizontal gene transfer (HGT) by MGEs can promote the ready dissemination of ARGs among microbial communities (Xie et al., 2018a). Furthermore, previous studies assumed that environmental ARGs could be transferred into the food chain through HGT mechanisms, creating great risks to human health (Berendonk et al., 2015; Wang et al., 2015). In China, over 3 billion tons of livestock manure is produced each year, and most manure is applied to farmlands after no or little treatment (Wang et al., 2006; You and Silbergeld, 2014). This practice might introduce antibiotics and ARGs into soils (Qiao et al., 2012) and further spread ARGs in the soil microbial community. The North China Plain (NCP) is one of the most important grain production areas in China. The excessive use of chemical fertilizers in this region has caused serious negative environmental effects, such as soil acidification, ammonia volatilization, and nitrate contamination in groundwater (Chen et al., 2018; Wang et al., 2018). Applying organic manure to replace some amount of conventional chemical fertilizer has been proven to be an effective approach on the NCP with respect to crop yield and soil carbon and nitrogen stocks (Gai et al., 2018). Nonetheless, considering the high levels of ARGs and ARB in manure, we still lack a comprehensive understanding of the impact of manure fertilization on the spread of ARGs in the soils of the cropping systems on the NCP.

Manure application not only has an impact on the soil resistome but, as demonstrated in previous studies, also influences soil microbial communities (Jechalke et al., 2014; Udikovic-Kolic et al., 2014). In the past few decades, microbes have spread globally due to anthropogenic activities (Zhu et al., 2017a), and ARGs with unprecedented diversity and abundance have spread to different environments (Zhu et al., 2017b). Moreover, ARGs were observed to be stable in the microbial community of a soil that regularly received manure application (Heuer and Smalla, 2007). In addition, chemical fertilizer application can induce significant changes in soil properties and microbial communities (Wang et al., 2018; Xie et al., 2018b). Therefore, the importance of investigating and controlling ARGs and microbial communities in agricultural soils cannot be understated. However, only a few studies to date have systematically assessed the distribution and transport of ARGs in soil under long-term manure and chemical fertilizer application. In the present study, high-throughput quantitative PCR technology combined with high-throughput sequencing technology was employed to explore the influence of the application of chemical fertilizer and pig manure on the resistome profile, MGEs, and microbial communities of soil. The microbial community and soil properties were investigated in order to explore key factors shaping the diversity and distribution of ARGs in farmland soils. This study contributes to a better understanding of the dissemination and persistence of soil ARGs caused by manure application and chemical fertilization.

## Materials and Methods

### Soil Sampling

The long-term field experiment was set up in 2001 at the Luancheng Ecological Station, Hebei Province, China (37°53’N, 114°41’E) with a winter wheat-summer maize rotation. In this study, six treatments were used as follows: (1) CK (control, no fertilizers were added); (2) pig manure (M); (3) pig manure and nitrogen fertilizer (MN); (4) nitrogen fertilizer (N); (5) nitrogen and phosphorous fertilizers (NP); and (6) nitrogen, phosphorous and potassium fertilizers (NPK). There were three replicates for each treatment (plot size 6 × 16 m) distributed at random in the experimental field. Detailed information about the experimental design and the fertilization scheme is shown in Supplementary Figures S1, S2, respectively. The amounts of chemical fertilizers and pig manure applied are shown in Table 1. Fresh manure was applied as a base fertilizer to the agricultural soil in the M and MN treatments before wheat sowing every year (early October). The pig manure properties were as follows: total N of 13.7 mg/kg, total P of 58.7 mg/kg, and total K of 26.6 mg/kg. In January 2016, soil samples (0 to 15 cm depth) were collected from a winter wheat field by taking five soil cores from each plot and mixing them for one composite sample. Sampling occurred in the winter, when the wheat plants were small and had basically stopped growing. A total of 18 soil samples collected from each plot were transported on ice to the laboratory. Each sample was divided into two parts: one part was air-dried and used to analyze soil properties, and the other part was stored at −80°C for molecular analysis. The basic soil properties of each treatment are listed in Supplementary Table S1. Fresh pig manure was also preserved for molecular and physiochemical analyses.

**TABLE 1:** Annual input rates of manure and chemical fertilizers in the different treatments.

**Treatment**	Pig manure*	**Urea**	**Superphosphate**	**Potassium chloride**
	
	**kg hm^–2^ (dry weight)**	**kg hm^–2^**	**kg hm^–2^**	**kg hm^–2^**
CK	0	0	0	0
N	0	300	0	0
NP	0	300	120	0
NPK	0	300	120	75
M	4000	0	0	0
MN	5500	300	0	0

*Fresh manure was applied as a basal fertilizer to agricultural soil before wheat sowing every year (early October).

### Soil Property Analysis

The soil properties were measured according to previously described methods (Lu, 1999). Briefly, soil organic matter (OM) was determined using the K_2_Cr_2_O_7_ oxidation method. Soil pH was measured in a 1:5 (soil/carbon dioxide-free water) suspension using a pH meter (FE28, METTLER TOLEDO, Switzerland). Soil available phosphorus (AP) was extracted with 0.5 M NaHCO_3_ and determined using the molybdenum blue method. Available potassium (AK) was extracted with 1 M ammonium acetate and measured using flame photometry (ZEENIT^®^700P, Analytik Jena AG, Germany). Total carbon (TC) and total nitrogen (TN) were assessed using an Element Analyzer (Vario PYDO cube, Elementar, United States).

### DNA Extraction

Soil DNA was extracted using the FastDNA Spin Kit for Soil (MP Biomedicals, United States) following the manufacturer’s protocol. The quality and quantity of the extracted DNA were examined using a Nanodrop spectrophotometer (NanoDrop^TM^ One, Thermo Fisher Scientific, United States). The DNA was stored at −20°C until further analysis.

### High-Throughput Quantitative PCR (HT-qPCR)

High-Throughput Quantitative PCR was performed to investigate the distribution of ARGs in soil samples using the SmartChip Real-time PCR system (Wafergen Inc., United States) as previously described (Wang et al., 2014b). Quality assurance/quality control (QA/QC) was applied based on the standard method of Wafergen Biosystems^1^. Briefly, 285 ARGs, 10 MGEs [namely 8 transposase genes, 1 universal class I integron-integrase gene (*intI1*), and 1 clinical class I integron-integrase gene (*cintI*)], and 1 16S rRNA gene were analyzed (Su et al., 2015; Supplementary Table S2). In addition, a negative control was included on each chip for each primer set. The reaction conditions were as follows: initial denaturation at 95°C for 10 min, followed by 40 cycles of denaturation at 95°C for 30 s and annealing at 60°C for 30 s. Melting curve analyses were performed automatically using Wafergen software. The HT-qPCR results were calculated using SmartChip qPCR Software (v 2.7.0.1). A threshold cycle (Ct) of 31 was set as the detection limit, and amplification with an efficiency range from 80 to 120% was considered valid amplification (Zhu et al., 2013; Wang et al., 2014b). The relative abundance of each ARG/MGE was calculated using a formula from a previous study (Chen Q. et al., 2016): relative gene abundance $=\frac{10^{\left(\left(31-C\,t_{\left(t\,a\,r\,g\,e\,t\right)}\right)/\left(10/3\right)\right)}}{10^{\left(\left(31-C\,t_{\left(16\,s\right)}\right)/\left(10/3\right)\right)}}$, where Ct_(target)_ and Ct_(__16__*S)*_ referred to the threshold cycles of the ARGs/MGEs and the 16S rRNA gene, respectively. The relative abundance of each ARG/MGE was transformed to absolute gene abundance by normalizing to the 16S rRNA gene copy numbers, which were quantified by the TaqMan probe method (Suzuki et al., 2000). Fold changes were calculated to illustrate the enrichment of soil ARGs under the application of manure or chemical fertilizers according to a previous study (Wang et al., 2014b). Genes were regarded as statistically enriched compared with the CK treatment if the range calculated by two standard deviation of the mean fold change was entirely >1.

### High-Throughput Sequencing of the Bacterial 16S rRNA Gene

To depict bacterial communities, the 341F/785R primer pair was used to amplify the V3-V4 region of the 16S rRNA gene (Yasir et al., 2015). Each 25 μl PCR mixture consisted of 12.5 μl Premix Ex Taq (Takara Biotechnology, Dalian, China), 0.5 μl each primer (10 μM), 1 μl DNA template and 10.5 μl sterilized water. The thermal cycle included an initial denaturation at 95°C for 3 min, followed by 23 cycles of denaturation at 95°C for 30 s, annealing at 55°C for 30 s and extension at 72°C for 30 s, with a final extension for 5 min at 72°C. A subsequent 8-cycle PCR was performed to add the sequencing adapter and dual-index barcodes after the PCR products were purified. The final PCR products were sequenced using the Illumina MiSeq platform (Illumina, San Diego, CA, United States) at Shanghai Jiao Tong University. The obtained raw sequences were used to generate clean sequences according to our previous methods (Chen et al., 2018). The clean data generated were analyzed using Quantitative Insights Into Microbial Ecology (QIIME) software. Operational taxonomic units (OTUs) were determined at the 97% similarity level using the UCLUST program (Edgar, 2010). Taxonomy assignment was performed with the Greengenes database (DeSantis et al., 2006). The sequencing data were deposited in the European Nucleotide Archive database under accession number PRJEB29291.

### Statistical Analysis

A combination of one-way analysis of variance (ANOVA) and Duncan’s test was performed to compare the significance of differences among samples using SPSS 13.0 at a *P* < 0.05 significance level. Pearson correlation analysis and box plots were also generated using SPSS 13.0. Bar charts and scatter diagrams were generated by SigmaPlot 12.5. Circos graphs were produced online using Circos software^2^. Heatmap graphs were drawn using R (version 3.1.0) with the gplots package^3^. Redundancy analysis (RDA), variation partitioning analysis (VPA), and principal coordinate analysis (PCoA) based on Bray-Curtis distance were performed using R with the “vegan” package (Oksanen et al., 2017). Ternary and Venn diagrams were constructed in R using the packages “vcd” and “gplots”, respectively. Network analysis was conducted using Cytoscape 3.6.0 based on the correlation matrix by calculating each pairwise Spearman’s rank correlation generated from R (“psych” package).

## Results

### Soil Properties

In this study, the long-term application of chemical fertilizers (N, NP, and NPK) significantly reduced soil pH (*P* < 0.05), while the increases in the soil OM, TC, TN, and AP were not significant (except that of AP in the NP and NPK treatments). Compared to the chemical fertilizer treatments, the application of manure fertilizer significantly decreased the pH and the C/N ratio and increased the concentrations of OM, TC, TN, AK, and AP (*P* < 0.05) (Supplementary Table S1).

### Diversity and Abundance of ARGs

A total of 108 unique ARGs amplified by 137 ARG primer sets were detected in pig manure (PM). Tetracycline (19%) and macrolide-lincosamide-streptogramin B (MLSB) (19%) resistance genes were the most abundant resistance genes, and antibiotic deactivation (43%) was the main resistance mechanism. The total relative abundance of ARGs was 1.31 (±0.21) copies/16S rRNA gene copies in the PM (Supplementary Table S3).

**FIGURE 1:**
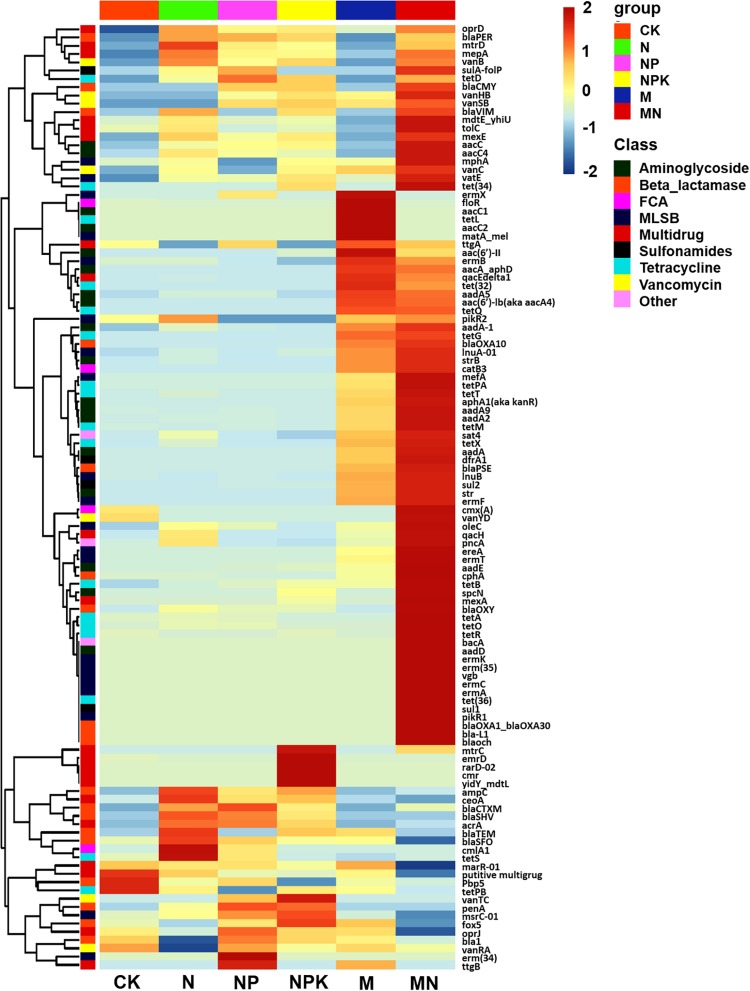
Heatmap graph showing the profiles of ARGs in the different treatments. Each row represents a single ARG, and each column represents the soil treatment. Data were standardized using the absolute abundance of ARGs. FCA, fluoroquinolone, quinolone, florfenicol, chloramphenicol, and amphenicol; MLSB, macrolide-lincosamide-streptogramin B.

**FIGURE 2:**
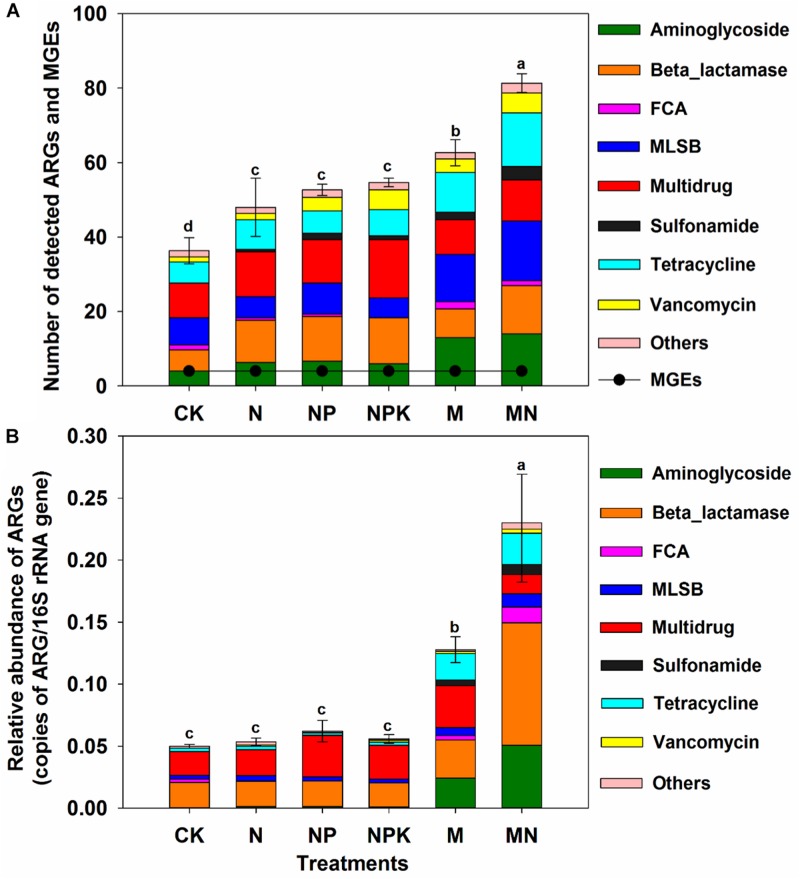
**(A)** Number of detected ARGs and MGEs in the different treatments. **(B)** The relative abundance of ARGs in the different treatments. FCA, fluoroquinolone, quinolone, florfenicol, chloramphenicol, and amphenicol; MLSB, macrolide-lincosamide-streptogramin B; MGEs, mobile genetic elements. Different letters indicate significant differences of means in pairwise comparisons (Duncan’s test, *P* < 0.05) for each treatment.

**FIGURE 3:**
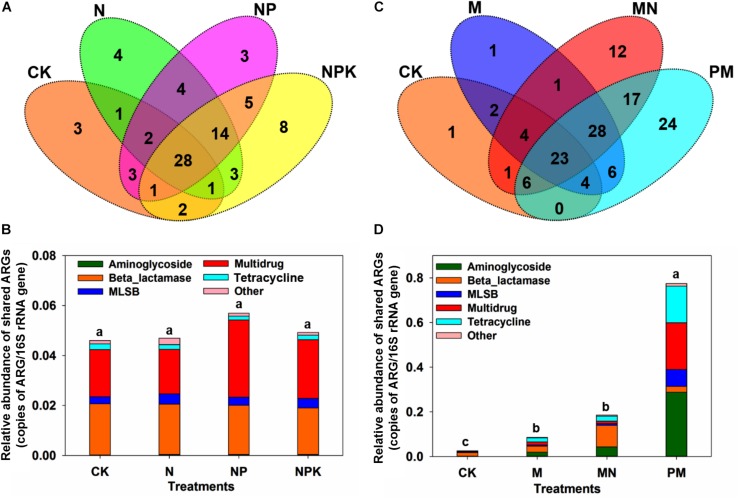
**(A)** Venn diagram illustrating the ARGs shared by the CK, N, NP, and NPK treatments. **(B)** The relative abundance of ARGs shared among the CK, N, NP, and NPK treatments. **(C)** Venn diagram illustrating the ARGs shared by the CK, M, and MN treatments and PM. **(D)** The relative abundance of ARGs shared among the CK, M, and MN treatments and PM. MLSB, macrolide-lincosamide-streptogramin B. Different letters indicate significant differences of means in pairwise comparisons (Duncan’s test, *P* < 0.05) for each treatment.

A total of 114 unique ARGs amplified by 152 ARG primer sets were detected in all soil samples (Figure 1). The 114 ARGs detected in soil samples potentially encoded resistance to major types of antibiotics, including multidrug antibiotics (18%), MLSB (18%), aminoglycoside (16%), beta_lactamase (17%), tetracycline (15%), FCA (fluoroquinolone, quinolone, florfenicol, chloramphenicol and amphenicol) (3%), vancomycin (6%), and sulfonamides (4%) (Supplementary Figure S3A), covering three major resistance mechanisms: antibiotic deactivation (39%), efflux pump (31%) and cellular protection (27%) (Supplementary Figure S3B). The detection frequency ranged from 36 to 81, with MN treatment having the highest detection frequency (Figure 2A). The Shannon index demonstrated that the application of chemical fertilizer and manure increased the diversity of ARGs, with manure-fertilized treatments having much higher diversity (Supplementary Figure S4A). Compared with the CK and chemical-fertilized treatments, manure application significantly increased the ARG abundance, with total relative abundances of ARGs in the M and MN of 0.13 (2.6 times) and 0.23 (4.6 times) copies/16S rRNA gene copies, respectively (Figure 2B). The higher abundance of ARGs detected in manure-fertilized treatments was mainly due to four types of ARGs: aminoglycoside, beta_lactamase, multidrug and tetracycline resistance genes (Supplementary Figure S5). For example, the relative abundances of aminoglycoside resistance genes were 0.02 and 0.05 copies/16S rRNA gene copies in the M and MN treatments, respectively, 86 and 181 times higher than in the CK treatment (Supplementary Table S3). Notably, sulfonamide resistance genes became detectable in chemical- and manure-fertilized soils. According to the Venn diagrams, 28 ARGs were shared by the CK and chemical-fertilized treatments (Supplementary Table S4); 25, 28 and 30 unique ARGs were detected in the N, NP, and NPK treatments, respectively (Figure 3A). Twenty-three ARGs were shared by the CK, manured soils and pig manure (PM) (Supplementary Table S5); 36 and 58 unique ARGs were detected in the M and MN treatments, respectively (Figure 3C). In addition to providing additional new ARGs, manure application significantly increased the abundance of indigenous resistance genes compared with that in the chemical fertilization treatments (Figures 3B,D).

The PCoA based on Bray-Curtis distance showed that the ARG distributions in the CK treatment and fertilized soil samples were separated (Supplementary Figure S4B). The ARG profile of the CK treatment showed obvious differences from the M and MN treatments on the first axis, which explained 73.6% of the variance (Supplementary Figure S4B); a significant difference from the chemical-fertilized treatments (N, NP, and NPK) was also observed on the second axis, which explained 11.1% of the variance (Supplementary Figure S4B). These results indicated that fertilization could alter the overall distribution of ARGs. In addition, the changes in soil properties caused by fertilization might contribute to the structural variances in ARGs. RDA showed that the selected soil properties explained a total of 47.9% of the structural variance in ARGs. Among them, the pH and the C/N ratio were negatively correlated with the abundance of ARGs in soil, whereas other soil properties, such as TC, OM, TN, AK, and AP, were positively correlated with ARG abundance (*P* < 0.05) (Supplementary Figure S6).

### Enrichment of ARGs

**FIGURE 4:**
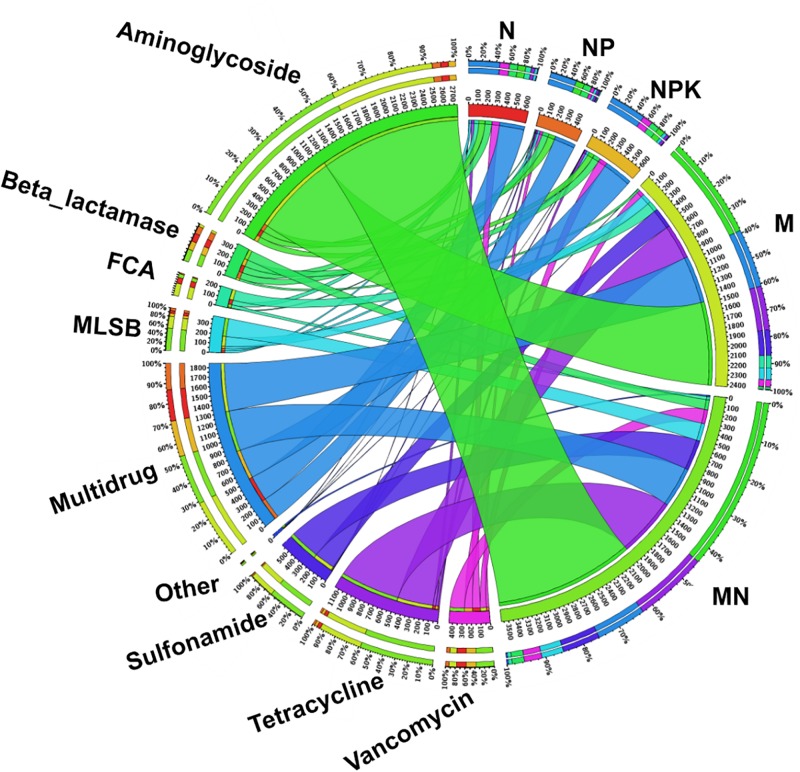
The distribution of each ARG subtype in fertilized soils determined using Circos software (http://circos.ca/). Numbers on the inner ring represent the total fold change of each fertilization treatment. Numbers on the outer ring represent the percentages of ARG subtypes in each fertilization treatment. FCA, fluoroquinolone, quinolone, florfenicol, chloramphenicol, and amphenicol; MLSB, macrolide-lincosamide-streptogramin B.

The fold change in ARGs compared with the levels in the CK treatment was assumed to represent ARG enrichment in each chemical- and manure-fertilized sample. The total enrichment of ARGs in fertilized soils displayed marked differences, ranging from 482.1-fold (NP) to 3574.9-fold (MN) compared with the CK treatment (Figure 4). Genes encoding resistance to aminoglycoside, multidrug and tetracycline were the three most dominant types of ARGs in the fertilized treatments. According to the fold change values of each subtype of ARG (Supplementary Table S6), *aadA2-03* (421.3-fold, MN), *tetG-02* (259.1-fold, MN), *sul2* (303.8-fold, MN), *ermF* (54.0-fold, MN), *floR* (94.6-fold, M), *ampC-04* (27.6-fold, N), and *vanC-03* (98.6-fold, MN), encoding resistance to aminoglycoside, tetracycline, sulfonamide, MLSB, FCA, beta_lactamase, and vancomycin, respectively, exhibited the highest enrichment. Additionally, up to 134 ARGs were statistically enriched in at least one soil sample, with statistical enrichment values ranging from 426.7- to 3542.8-fold (Supplementary Figure S7). The application of manure led to a significantly higher enrichment of ARGs than the application of chemical fertilizer.

### Cooccurrence Among ARG Subtypes

The cooccurrence patterns among ARG subtypes were evaluated through network analysis, which indicated a significant correlation within the networks (Spearman’s correlation coefficient *r* > 0.6, *P* < 0.01) (Supplementary Figure S8). This network included 60 nodes and 212 edges, with nodes covering all major types of ARGs and MGEs. A total of five modules were formed; module I and module II were the two largest modules, with intensive connections within each module. The most intensively connected node was defined as the “hub” gene in each module. For instance, the *tetX* gene, which encodes resistance to tetracycline, was the hub gene of module I and had direct connections to 18 genes. In module II, *blaOXY*, which encodes resistance to beta_lactamase, was the hub gene and had direct connections to 13 genes. The other three modules only contained 2 to 3 ARGs.

### Diversity and Abundance of MGEs

**FIGURE 5:**
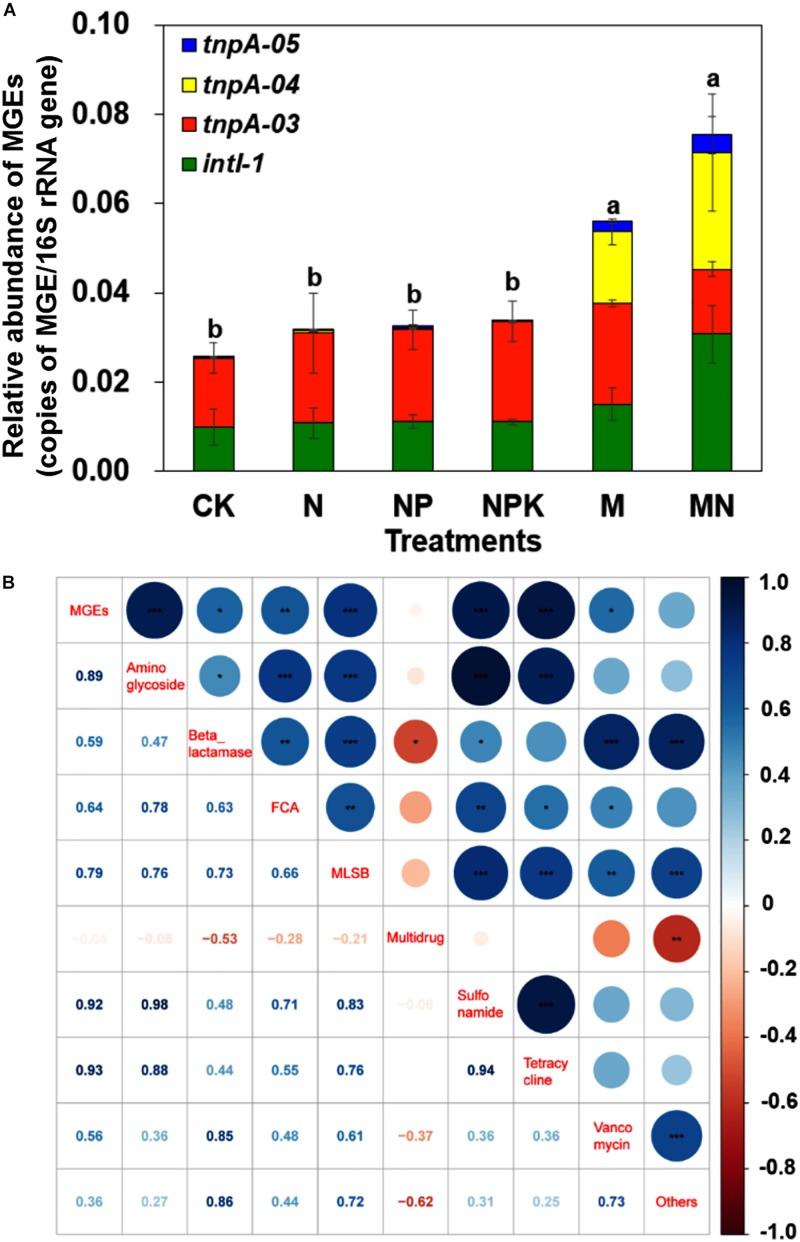
**(A)** The relative abundance of MGEs in different treatments. **(B)** Pearson correlation coefficients of the relative abundances of ARGs and MGEs across all treatments. Different letters indicate significant differences of means in pairwise comparisons (Duncan’s test, *P* < 0.05) for each treatment.

Eight MGEs, including seven transposase genes and one integrase gene, were detected in pig manure, with a total relative abundance of 0.12 copies/16S rRNA gene copies (Supplementary Table S3). In addition, *tnpA*-03, *tnpA*-04, *tnpA*-05 and *intI* 1 genes were detected in all soil samples, with a total relative abundance ranging from 2.56 × 10^–2^ to 6.66 × 10^–2^ copies/16S rRNA gene copies (Figure 5A). Manure application significantly increased the relative abundance of MGEs, especially *tnpA*-04, though chemical fertilizer did not significantly increase the relative abundance of MGEs. Additionally, the total relative abundance of MGEs was significantly correlated with the total relative abundances of aminoglycoside (*r* = 0.89, *P* < 0.001), beta_lactamase (*r* = 0.59, *P* < 0.05), FCA (*r* = 0.64, *P* < 0.01), MLSB (*r* = 0.79, *P* < 0.001), sulfonamide (*r* = 0.92, *P* < 0.001), tetracycline (*r* = 0.93, *P* < 0.001), and vancomycin (*r* = 0.56, *P* < 0.05) resistance genes, based on Pearson’s correlation analysis (Figure 5B). Furthermore, as typical transposase genes, *tnpA-03*, *tnpA-04* and *tnpA-05* cooccurred with 2, 4, and 1 ARG subtypes, respectively. For example, *tnpA-04* showed strong correlations with *aadA2*, *aadA9*, *tetG*, and *tetX* genes, and the *intI1* gene had a strong relationship with 4 ARG subtypes: *aadE*, *aadA2*, *sul2*, and *tetX* (Supplementary Figure S8).

### Microbial Communities

The absolute abundance of the bacterial 16S rRNA gene ranged from 1.52 to 4.19 × 10^10^ copies per gram of soil (dry weight, dw), with the highest abundance in manure-fertilized treatments (Supplementary Figure S9).

A total of 224,968 high-quality 16S rRNA gene sequences were obtained from all soil samples and clustered into 29,994 OTUs at 97% similarity, with an average of 4,316 OTUs per soil sample. The application of chemical fertilizer significantly reduced the alpha diversity of the soil microbial community, whereas manure amendment significantly increased the alpha diversity (Supplementary Figure S10A). Differences in the composition of microbial communities among the CK, manure-fertilized and chemical-fertilized treatments were also clearly identified (Supplementary Figure S11). The PCoA based on Bray-Curtis distance revealed clear separations (Adonis test, *P* < 0.01) between the manure-fertilized treatments (M and MN) and the CK treatment and between the chemical-fertilized treatments (N, NP, and NPK) and the CK treatment (Supplementary Figure S10B). The soil properties, i.e., TC, TN, OM, AK, AP, pH, and C/N ratio, were important factors in the separation of the microbial communities, explaining a total of 35.9% of the structural variance in the microbial community (Supplementary Figure S12).

### Correlation Analysis Between ARGs and Microbial Taxa

**FIGURE 6:**
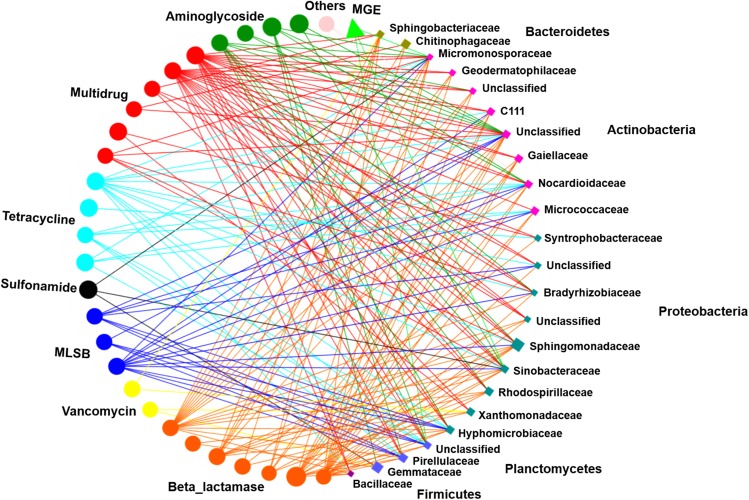
Correlations between ARG subtypes and bacterial taxa were processed by network analysis. Each node was colored based on ARG type and microbial taxa. Each connection indicates a strong (Spearman’s correlation coefficient *r*^2^ > 0.36) and significant (*P* < 0.01) correlation. Edges are dependent on the coefficient values, and node size is weighted based on the relative abundance of ARGs/bacterial taxa. MLSB, macrolide-lincosamide-streptogramin B.

Network analysis was performed to illustrate the correlations between ARG subtypes and bacterial taxa (at the family level). The resulting network was composed of 52 nodes and 196 edges, including 28 unique ARG subtypes, 1 *intI1* gene, and 23 families (Figure 6). These 23 bacterial taxa were affiliated with the phyla Bacteroidetes, Actinobacteria, Proteobacteria, Planctomycetes, and Firmicutes and had strong correlations with various ARGs (Spearman’s *r*^2^ > 0.36, *P* < 0.01). At the family level, Micromonosporaceae were significantly correlated with 2 aminoglycoside resistance genes, 4 beta_lactamase resistance genes, 1 MLSB resistance gene, 2 multidrug resistance genes, 2 tetracycline resistance genes, 1 sulfonamide resistance gene, and 1 MGE. Sinobacteraceae displayed significant correlations with 2 aminoglycoside resistance genes, 4 beta_lactamase resistance genes, 3 multidrug resistance genes, 1 MLSB resistance gene, 1 sulfonamide resistance gene, 1 tetracycline resistance gene, and 1 MGE. In addition, other families, such as Nocardioidaceae, Sphingomonadaceae, and Pirellulaceae, also had significant correlations with diverse ARGs (*P* < 0.01) (Figure 6).

### Relationships Among Soil Properties, Microbial Communities, MGEs, and ARGs

**FIGURE 7:**
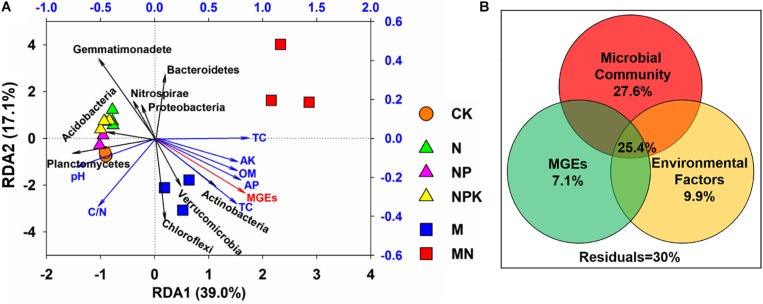
**(A)** Redundancy analysis (RDA) revealing correlations among ARGs, main phyla taxa (>1% in any sample) and soil properties. **(B)** Variation partitioning analysis (VPA) interpreting the effects of microbial community, soil properties and MGEs on the variations in ARGs. The absolute abundance of ARGs and MGEs was log10-transformed. The structure of the microbial community was represented using PCo1 and PCo2 from the PCoA based on Bray-Curtis distance.

Redundancy analysis was employed to evaluate the relationships among the microbial community, soil properties, and ARGs (Figure 7A). The selected variables explained 56.1% of the variance in the ARGs. Three phyla (Actinobacteria, Planctomycetes, and Gemmatimonadetes) exhibited significant correlations with ARGs in soil (*P* < 0.05). Actinobacteria showed a positive correlation with the first axis in the M treatment, while Planctomycetes and Gemmatimonadetes showed significant correlations with the second axis in the N, NP, NPK, and CK treatments. In addition, VPA was applied to explore the effects of the microbial community, MGEs, and soil properties on the distribution of ARGs (Figure 7B) and illustrated that the microbial community, MGEs and soil properties contributed to 27.6, 7.1, and 9.9% of the ARG variation, respectively. In addition, the interactions among the microbial community, MGEs and soil properties explained 25.4% of the variation in ARGs, which was higher than the explanatory power of soil properties (9.9%) and MGEs (7.1%).

## Discussion

### The Diversity and Abundance of ARGs in Manure-Fertilized Soils

After 15 years of continuous manure amendment, the diversity and abundance of ARGs were significantly increased in manure-fertilized soil (Figure 2), and the antibiotic deactivation and efflux pump mechanisms were the two most dominant resistance mechanisms (Supplementary Figure S3). This was in line with previous studies (Zhang et al., 2011; Joy et al., 2013; Yang et al., 2013; Fahrenfeld et al., 2014; Chen et al., 2017). The PCoA also showed that manure application influenced the ARG composition (Supplementary Figure S4). Previous studies have shown that manure application correlates with the emergence and proliferation of ARGs in indigenous microbiota (Su et al., 2014; Chen Q. et al., 2016). In addition, manure fertilizer mainly enhanced the abundance of shared ARGs between the manure-fertilized treatment and the CK treatment, such as aminoglycoside and tetracycline resistance genes. It is possible that the shared ARGs are sustained and disseminated more effectively in long-term manured soils. On the other hand, the elevated nutrient inputs provided by manure addition, such as soil OM, can stimulate the growth of the soil microbial community and induce a bloom of some native ARB (Udikovic-Kolic et al., 2014). Moreover, additional new ARGs were also introduced into fertilized soil by manure application, as 36 and 58 unique ARGs were detected in the M and MN treatments, respectively, but not in the CK treatment (Figure 3). This may have been caused by the introduction of new types of ARGs and ARB from animal manure (Heuer et al., 2011; Chen et al., 2017; Pu et al., 2018) or by selection pressure forced by antibiotics, heavy metals and disinfectants in the manure-fertilized soils (Knapp et al., 2010; Su et al., 2014; Udikovic-Kolic et al., 2014; Xie et al., 2018a). A previous study demonstrated that selective agents played important roles in transferred gene persistence in the new host and in disseminating the newly emerged genes (Chee-Sanford et al., 2009; Bengtsson-Palme and Larsson, 2015).

The different ARGs detected showed various levels of enrichment, which likely depended not only on antibiotic selection but also on the relative growth and decay of hosts as well as the ability of ARGs to undergo HGT. Moreover, as indicated by the network analysis, genes that encoded resistance to different types of antibiotics were placed in the same module (Supplementary Figure S8); they might change and transfer together under the selection pressure imposed by manure application because these ARGs may be located on the same DNA fragment or in the same host bacterium (Qian et al., 2018). Previous studies also found cooccurrence among different ARGs (Li et al., 2015; Johnson et al., 2016; Chen et al., 2017; Zhang et al., 2017; Zhu et al., 2017b), suggesting that cooccurrence patterns are widespread in diverse environments.

### The Diversity and Abundance of ARGs in Chemical-Fertilized Soils

The long-term application of chemical fertilizer only moderately augmented the diversity of ARGs but had no apparent effect on the abundance of ARGs (Figure 2). This result was consistent with that in previous studies (Chen Q. et al., 2016; Xie et al., 2018b). The variation in the ARG profile between chemical fertilization treatments and the CK treatment was likely caused by alterations in the soil properties and the microbial community composition, including ARB already in the soil (Forsberg et al., 2014; Xie et al., 2018b). For instance, soil pH was found to be a main factor driving the differences in the soil microbial community structure between soils with and without chemical fertilizer application (Xie et al., 2018b). Although the CK treatment in this study had a diverse array of ARGs, the number and relative abundance of intrinsic resistance gene was significantly lower than that in manure-fertilized treatments. The existence of ARGs in unfertilized soil has traditionally been explained by the presence of antibiotic producers that harbor genes to protect themselves from these secondary metabolites (Heuer et al., 2011). Previous studies have also shown that antibiotic resistance is an ancient phenomenon, as confirmed by the detection of ARGs in permafrost sediments (D’Costa et al., 2011), pristine forests (Zhu et al., 2013; Wang et al., 2014a), and the Tibetan environment (Chen B. et al., 2016).

### Horizontal Gene Transfer Is an Essential Driver of ARG Dissemination

In this study, many types and a high abundance of MGEs were found in pig manure. Only manure amendment dramatically increased the relative abundance of MGEs in soil, while chemical fertilizer did not (Figure 5), indicating that MGEs originating from manure-derived bacteria could be established well in manured soils (Heuer et al., 2011). HGT of ARGs via MGEs, such as integrons, transposons, interactive conjugative elements and plasmids, from manure to soil microbes is a potential pathway for the spread and propagation of ARGs (Stokes and Gillings, 2011) since some of the bacteria from manure might not be well adapted to soil and may only survive from weeks to months in the environment (Heuer et al., 2011). HGT of ARGs from manure bacteria to indigenous soil bacteria may mediate ARG persistence in soil, which might be permanent and have unpredictable consequences for the entire soil microbiome (Gillings and Stokes, 2012). In addition, the relative abundances of total ARGs or of specific types of ARGs had significant correlations with those of total MGEs, as well as the significant cooccurrence among diverse ARGs and MGEs, meaning that ARGs were closely linked to MGEs. A previous study demonstrated that ARGs could undergo “mobilization” when they appeared on MGEs (Stokes and Gillings, 2011). For example, integrons commonly contained the resistance cassette *aadA* gene, as well as the *qacE*Δ*1* and *sul2* genes (Stokes and Gillings, 2011; Ma et al., 2017), which were also among the most enriched genes in this study. Tetracycline resistance genes are always located on transposons and other MGEs (Chopra and Roberts, 2001), the *floR* gene is located on the novel tnfloR transposon (Doublet et al., 2005), the *ermA* gene is located on transposon Tn554, and the *ermB* gene is located on transposon Tn551 (Saribas et al., 2006). Subsequently, ARGs located on MGEs could enhance the potential capacity of ARGs to survive in various environments through HGT events.

From a distance, manure-fertilized soil could be assumed to be a potential recruitment pool for ARGs. Manure application has been demonstrated to promote HGT of ARGs in soil, which may be partly induced by selection pressure in manure-fertilized soil (Gillings et al., 2009; Stokes and Gillings, 2011; Gillings, 2013). Antibiotic exposure increased the mobilization and transfer of ARGs (Bengtsson-Palme et al., 2018); thus, a manure-fertilized soil environment poses a high risk for the dissemination of ARGs. However, even in the absence of direct selection pressure from antibiotics, mobile ARGs may be favored by co-selection by other substances present, such as metals and biocides (Baker-Austin et al., 2006), as the resistance determinants for some of these compounds can be colocalized to the same MGEs as ARGs (Pal et al., 2015). Even though HGT by itself is probably not the main factor explaining the variation in ARGs in the fertilized soils (contributing to 7.1% of the ARG variation), the role of HGT in the spread of ARGs in the environment should not be neglected or underestimated; in particular, substantial numbers and abundances of ARGs and MGEs exist in manure-fertilized soil.

### The Soil Microbial Community Is the Main Driver Shaping ARG Profiles

In this study, the five most abundant phyla in soils, Proteobacteria, Actinobacteria, Bacteroidetes, Planctomycetes and Firmicutes, were assumed to be possible bacterial hosts for ARGs, as revealed by the network analysis (Figure 6). These bacterial phyla have been recognized as important hosts for ARGs that encode resistance to multiple antibiotics in metagenomics analysis (Forsberg et al., 2014). Among these phyla, Actinobacteria show diverse antibiotic resistance, as they produce antibiotics, strongly promoting an increase and enrichment of the resistome (Su et al., 2015). A previous study demonstrated that manure application could induce a significant selective advantage for ARGs affiliated with the Micromonosporaceae family (Xiong et al., 2018). Another study showed that the genera Bacillus and Mycobacterium were possible bacterial pathogen hosts of ARGs that were enriched in a tetracycline-added soil treatment (Xia et al., 2019). Therefore, since these ARB could readily grow there, their eventual antibiotic exposure would be much more likely to contribute to the selection pressure for resistance during environmental dissemination. Although further research, such as metagenomic analysis (Chen B. et al., 2016; Han et al., 2018), is needed to verify the correlation between ARGs and their potential bacterial hosts, the results still partially indicate that bacteria may carry certain specific ARGs and possess different abilities to obtain and transfer ARGs (Udikovic-Kolic et al., 2014; Miller et al., 2016).

A pronounced difference in the diversity and structure of the microbial community was found between the manure-fertilized soils and the CK and chemical-fertilized treatments (Supplementary Figure S10). Multiple factors could result in this phenomenon, such as plant species, root growth, exudate production, and soil properties (Chaparro et al., 2014; Wang et al., 2015). Importantly, the variation in the microbial community played the main role in causing the shifts in the antibiotic resistome, rather than soil properties or MGEs (Figure 7B), since to the extent of the manure application, all soil microbes would have been exposed to selection for the resistance and mobilization of ARGs (Su et al., 2015; Chen Q. et al., 2016; Han et al., 2018). Previous studies have demonstrated that microbial community structure is closely related to the ARGs harbored in different environments (Forsberg et al., 2014; Johnson et al., 2016; Xie et al., 2018b). In addition, soil has been deemed an important reservoir of ARGs due to its complex microbial community and diverse antibiotic-producing microbes (Su et al., 2014), and the establishment of ARGs in the soil microbial community can be promoted by the periodic addition of manure (Heuer and Smalla, 2007).

## Conclusion

In summary, this study demonstrated that long-term manure application markedly enhanced the abundance and diversity of ARGs that encode resistance to a variety of antibiotics, such as aminoglycoside and tetracycline. The increased abundance and diversity of ARGs in manure-fertilized soil was mainly caused by the manure stimulating a bloom of indigenous ARGs and introducing new ARGs. The higher enrichment of ARGs and MGEs in manure-fertilized soils, combined with the significant and positive correlations between ARGs and MGEs, indicated that horizontal gene transfer might facilitate the dissemination of resistance in soil bacteria via MGEs. In addition, manure application significantly changed the soil microbial community, which was assumed to be the main driver shaping the soil ARG profile. In contrast, chemical fertilization only significantly altered the structure of the soil microbial community but had less impact on the soil ARG profile. Hence, the risk of the propagation of ARGs from manure application is a significant concern, and mechanisms and measures for controlling these risks need to be explored further.

## Data Availability Statement

The datasets generated for this study can be found in the European Nucleotide Archive database, PRJEB29291.

## Author Contributions

FW, WH, and SC collected the soil samples and performed the laboratory measurements and data analysis. WD and CH designed and managed the experimental field. FW, MQ, and BL wrote and revised the manuscript. All authors read and approved the final version of the manuscript.

## Conflict of Interest

The authors declare that the research was conducted in the absence of any commercial or financial relationships that could be construed as a potential conflict of interest.

## Footnotes

1 http://www.genengnews.com/gen-articles/high-throughput-nanovolume-qpcr/2993/?page=2

2 http://circos.ca/

3 https://cran.r-project.org/web/packages/gplots/index.html
