# Supplementary material for: Fifteen-Year Application of Manure and Chemical Fertilizers Differently Impacts Soil ARGs and Microbial Community Structure
Source: Front Microbiol. 2020 Feb 6;11:62. doi: 10.3389/fmicb.2020.00062 (PMC7015874; doi:10.3389/fmicb.2020.00062)
Supplement: Supplementary file 1 [file Data_Sheet_1.doc]

**Fifteen-year application of manure and chemical fertilizers differently impacts soil ARGs and microbial community structure**

Fenghua Wang^a†^, Wanxue Han^a,b†^, Shuaimin Chen^c^, Wenxu Dong^a^, Min Qiao^d^, Chunsheng Hu^a^, Binbin Liu^a*^

^a^ Key Laboratory of Agricultural Water Resources, Hebei Key Laboratory of Soil Ecology, Center for Agricultural Resources Research, Institute of Genetics and Developmental Biology, Chinese Academy of Sciences, Shijiazhuang 050021, China

^b^ University of Chinese Academy of Sciences, Beijing 100039, China

^c^ Institute of Agricultural Resource and Environment, Jilin Academy of Agricultural Sciences, Changchun 130033, China.

^d^ State Key Lab of Urban and Regional Ecology, Research Center for Eco-Environmental Sciences, Chinese Academy of Sciences, Beijing 100085, China

*Corresponding author: Dr. Binbin Liu (binbinliu@sjziam.ac.cn)

Tel.: +86 311 85817713; fax: +86 311 85815093

**Author Contributions**

† These authors contributed equally.

Article Type: Original Research

Number of Figures: 12

Number of Tables: 6


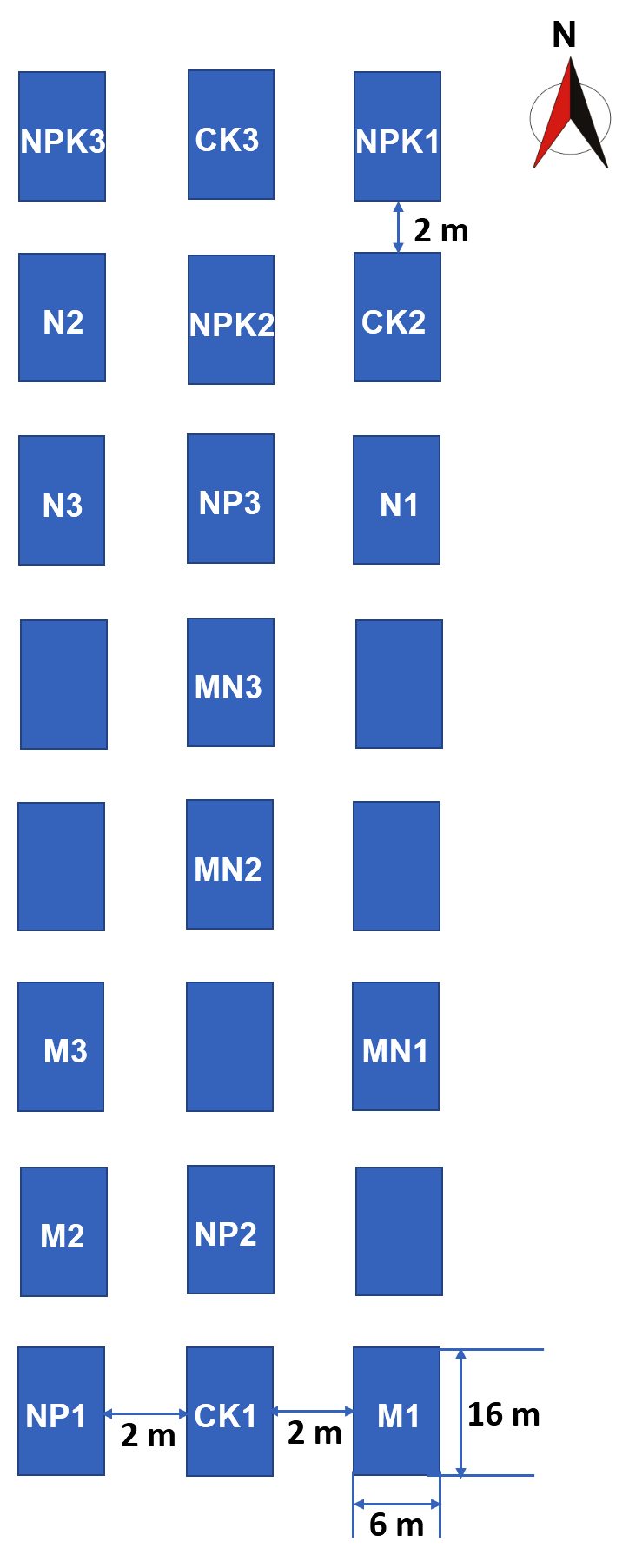


**Figure S1.** Sketch map of the plot distribution. The distance between groups was 2 m. Plot area is 96 m^2^ (6 m × 16 m)


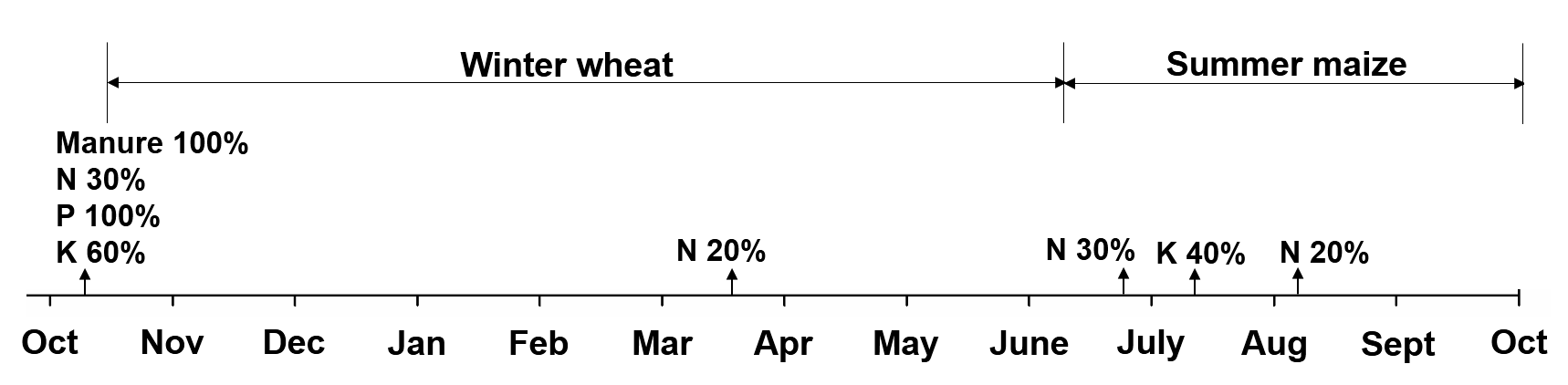


**Figure S2.** Fertilization regime in each year. Manure represents fresh pig manure; N represents urea fertilizer; P represents superphosphate fertilizer; K represents potassium chloride fertilizer.


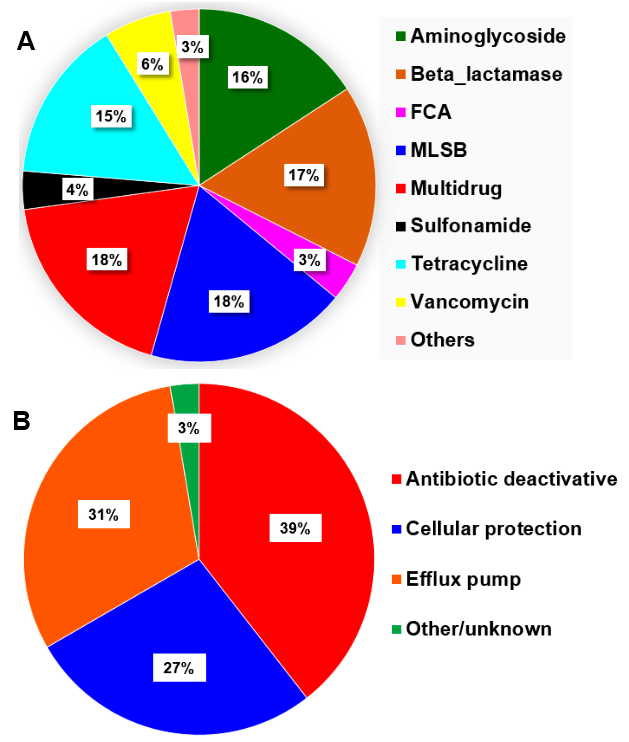


**Figure S3.** Resistance genes detected in all soil samples were classified based on (A) antibiotics to which they confer resistance and (B) mechanisms of resistance. FCA, fluoroquinolone, quinolone, florfenicol, chloramphenicol, and amphenicol; MLSB, macrolide−lincosamide−streptogramin B.


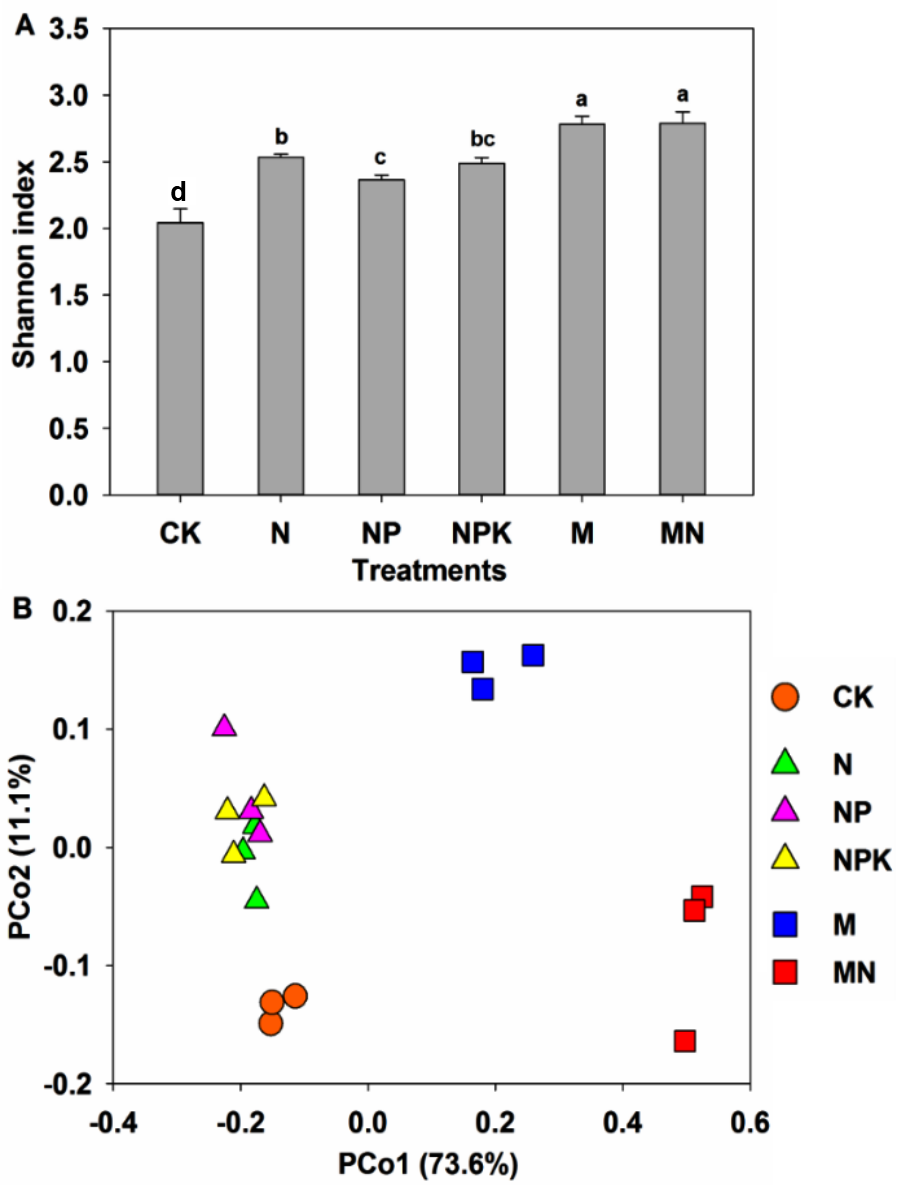


**Figure S4.** (A) Shannon index of soil ARGs. (B) Principal coordinates analysis of the soil ARGs (relative abundance of ARGs). Different letters indicate significant differences of means in pairwise comparisons (Duncan’s test; *P* < 0.05) for each treatment.


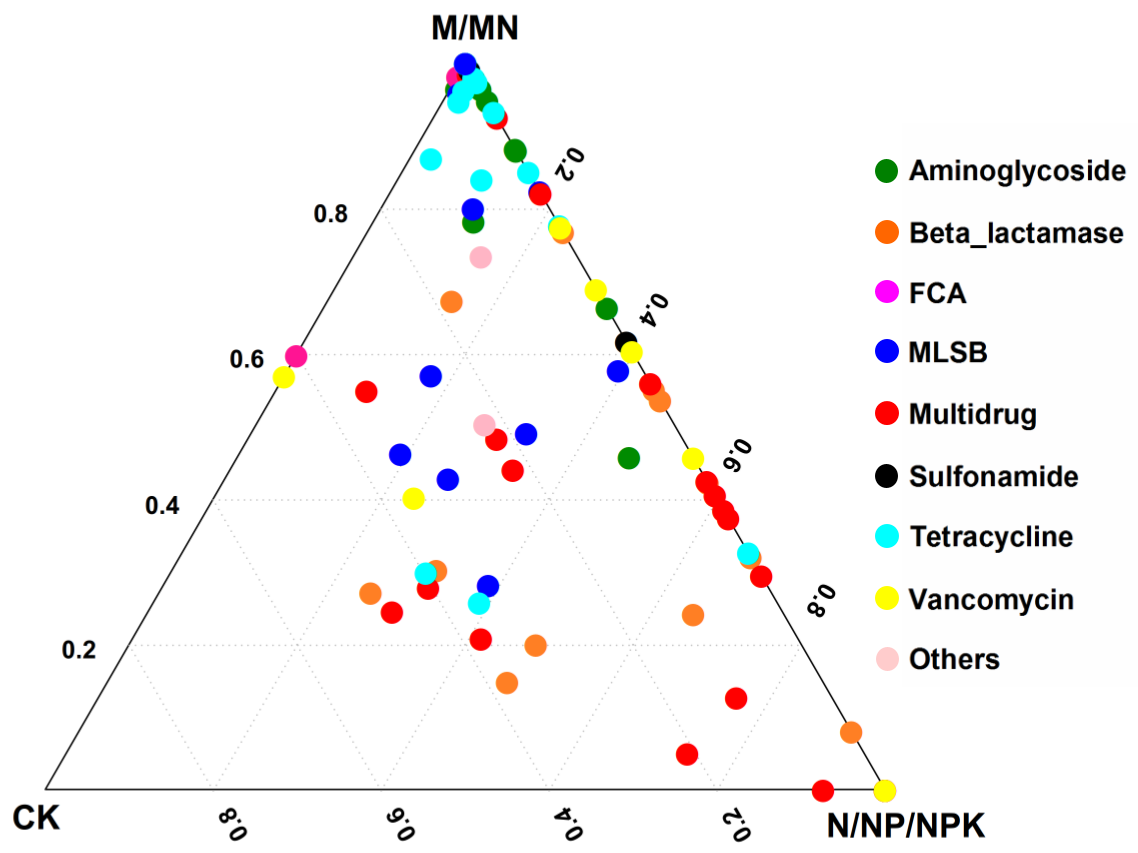


**Figure S5.** Ternary plot depicting ARGs shared between the control (left), chemical-fertilized soils (right), and manure-fertilized soils (top). FCA, fluoroquinolone, quinolone, florfenicol, chloramphenicol, and amphenicol resistance; MLSB, macrolide-lincosamide-streptogramin B; MGEs, mobile genetic elements.


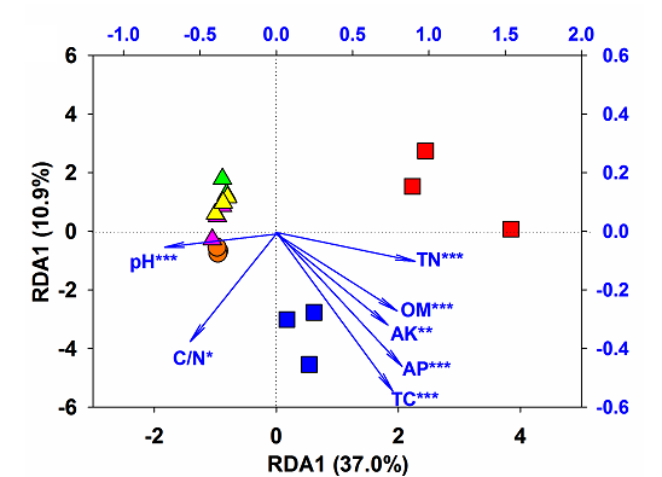


**Figure S6.** Redundancy analysis (RDA) assessed the contributions of soil properties to the structure of the ARG profiles. Soil properties including pH, C/N, TC, TN, OM, AK, and AP were used in the analysis. The variance inflation factor (vif) was used to check for the collinearity between parameters in the two analyses. Parameters showing a significant (*p* < 0.05) impact on profile structure are shown in the plots.


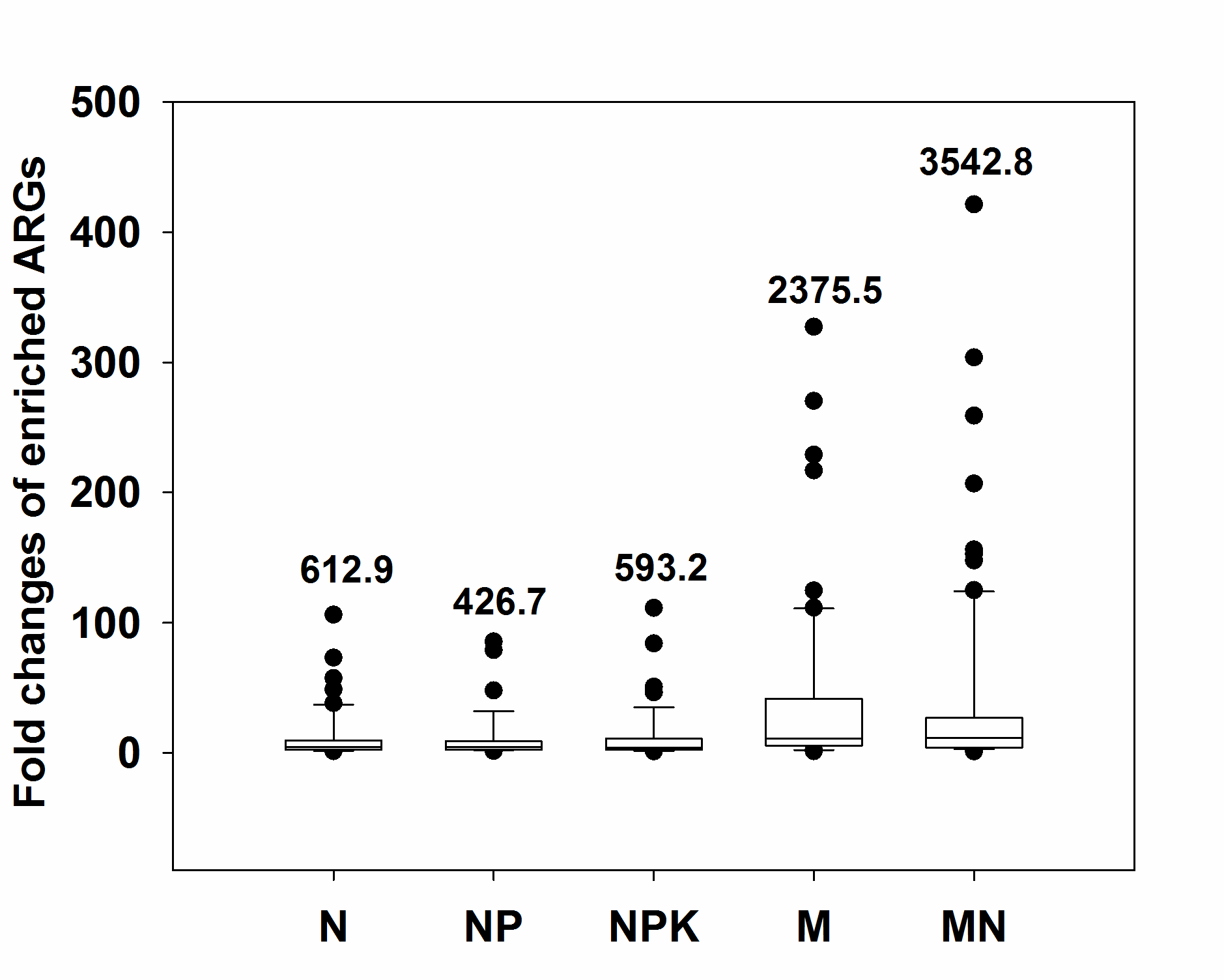


**Figure S7.** Statistically enriched resistance genes are shown in box plots. The number above each site indicates the sum of the numbers that yielded statistically significant results. The symbols indicate: box, 25th to 75th percentile; horizontal line, median; whiskers, 10th and 90th percentile.


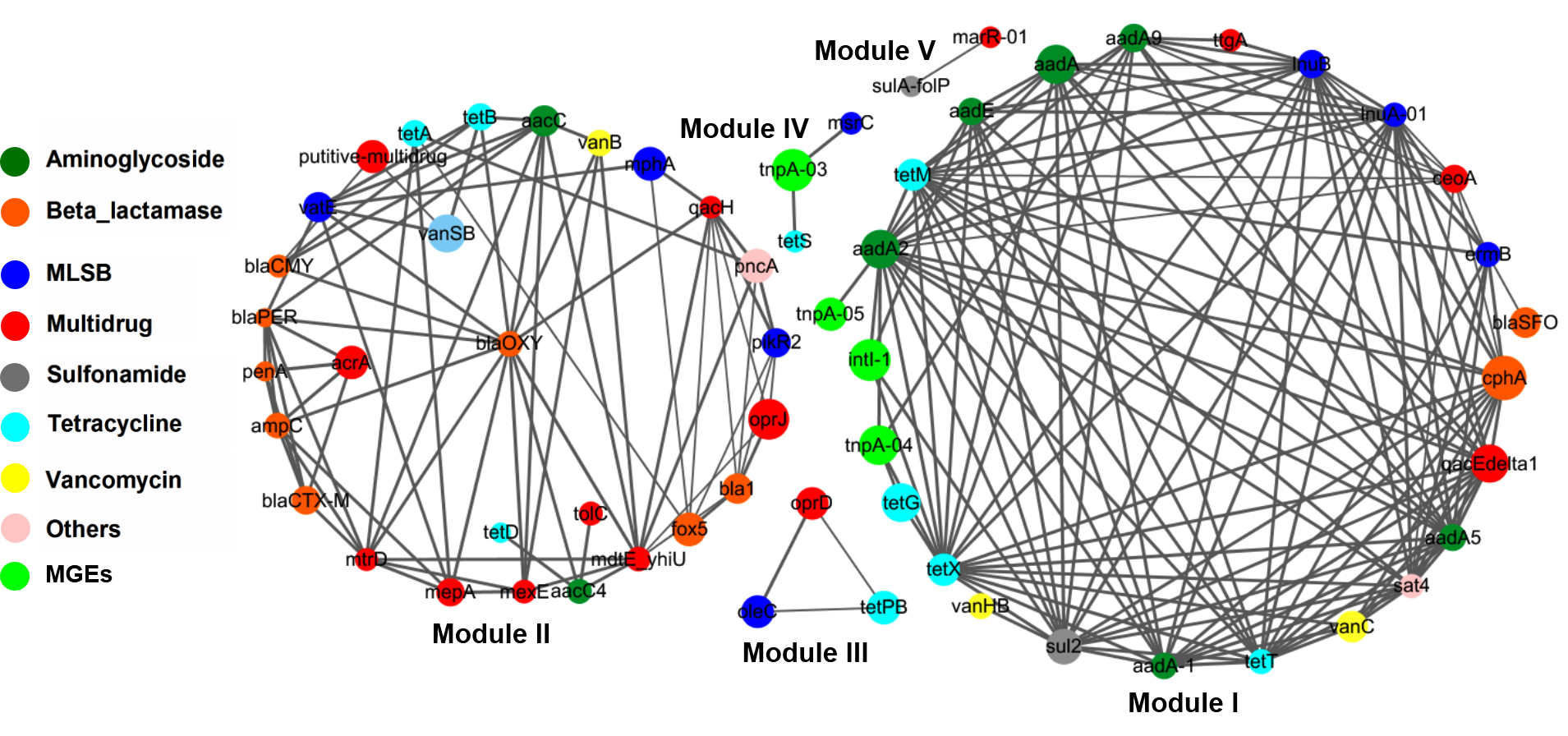


**Figure S8.** Network showing the correlations between ARG types and MGEs. Correlations with significant (*P* < 0.01) and strong (r ≥ 0.6) coefficients were retained for the network analysis. The edge width is dependent on the coefficient value. The node size is dependent on the average ARG abundance (lg10-transformed) in the detected samples.


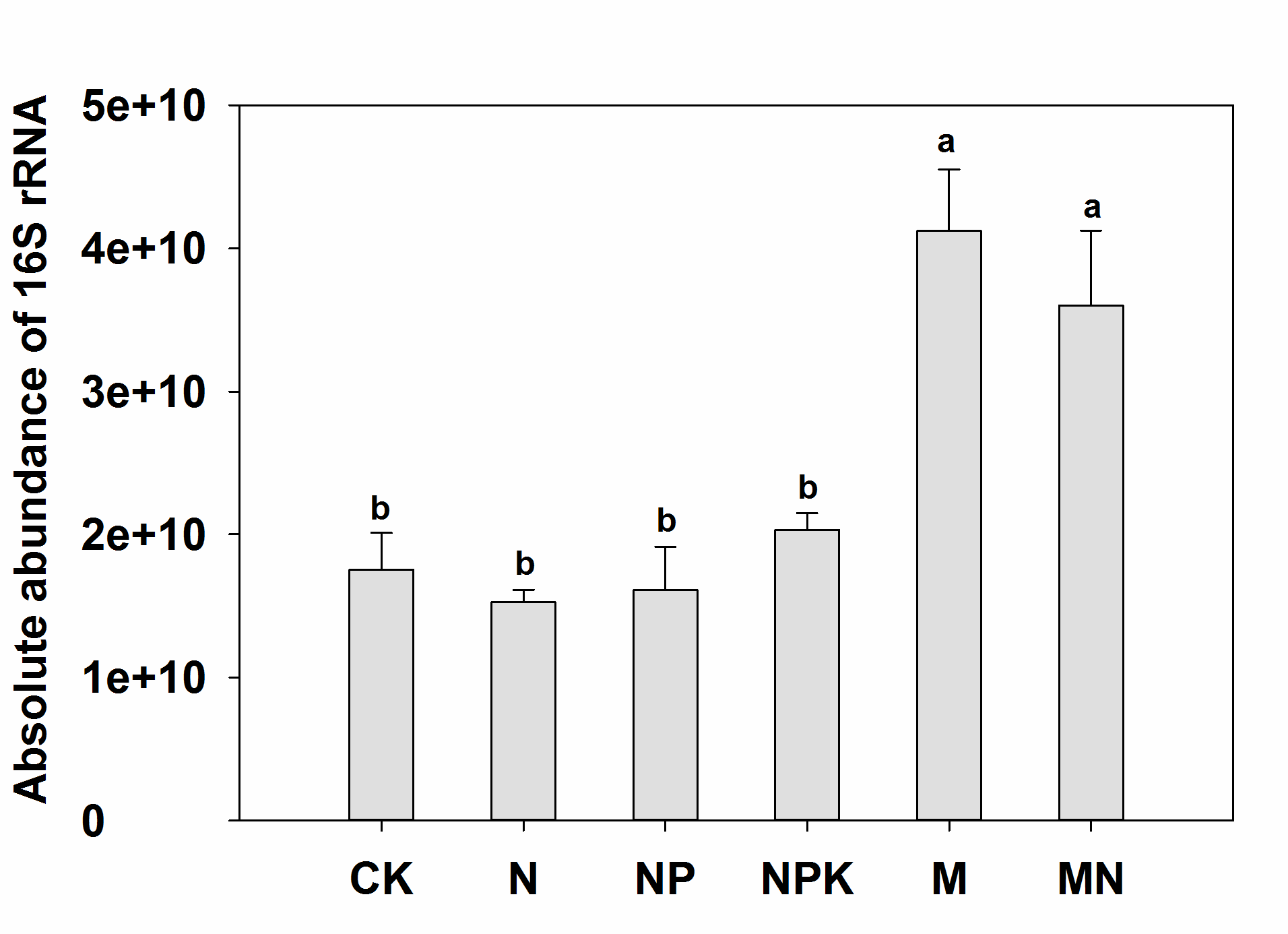


**Figure S9.** Absolute abundance of the 16S rRNA gene (copies per gram of dry soil) in treatments. Error bars represent the standard deviation (SD) of three replicates (n=3). Different letters indicate significant differences of means in pairwise comparisons (Duncan’s test; *P* < 0.05) for each treatment.


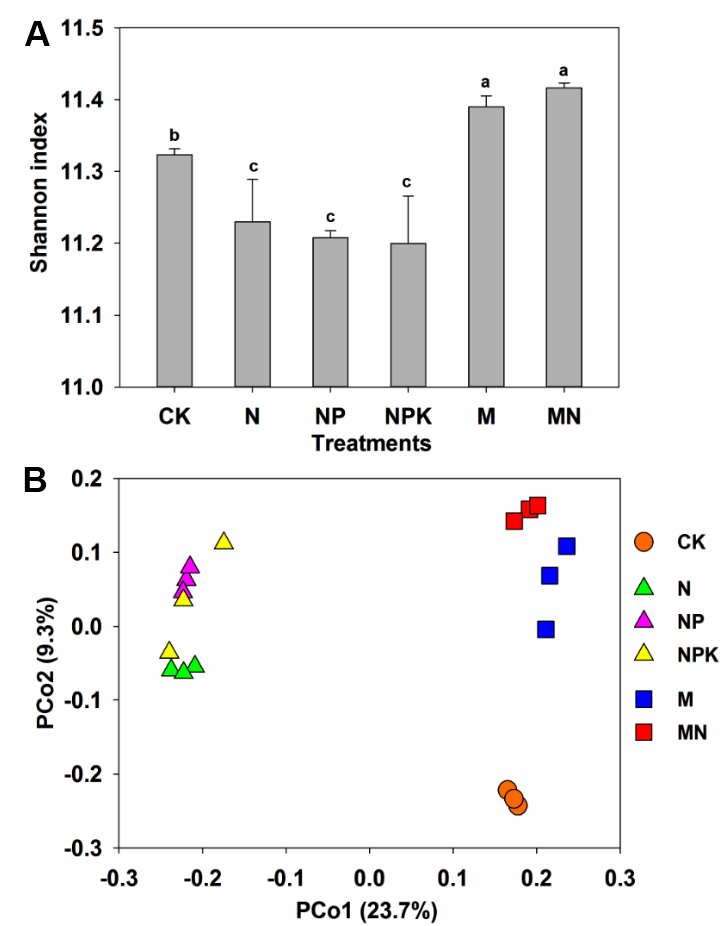


**Figure S10.** Diversity of the microbial community in soils. (A) Shannon index of soil ARGs. (B) Principal coordinate analysis (PCoA) of the soil bacterial community based on Bray-Curtis dissimilarity. Different letters indicate significant differences of means in pairwise comparisons (Duncan’s test; *P* < 0.05) for each treatment.


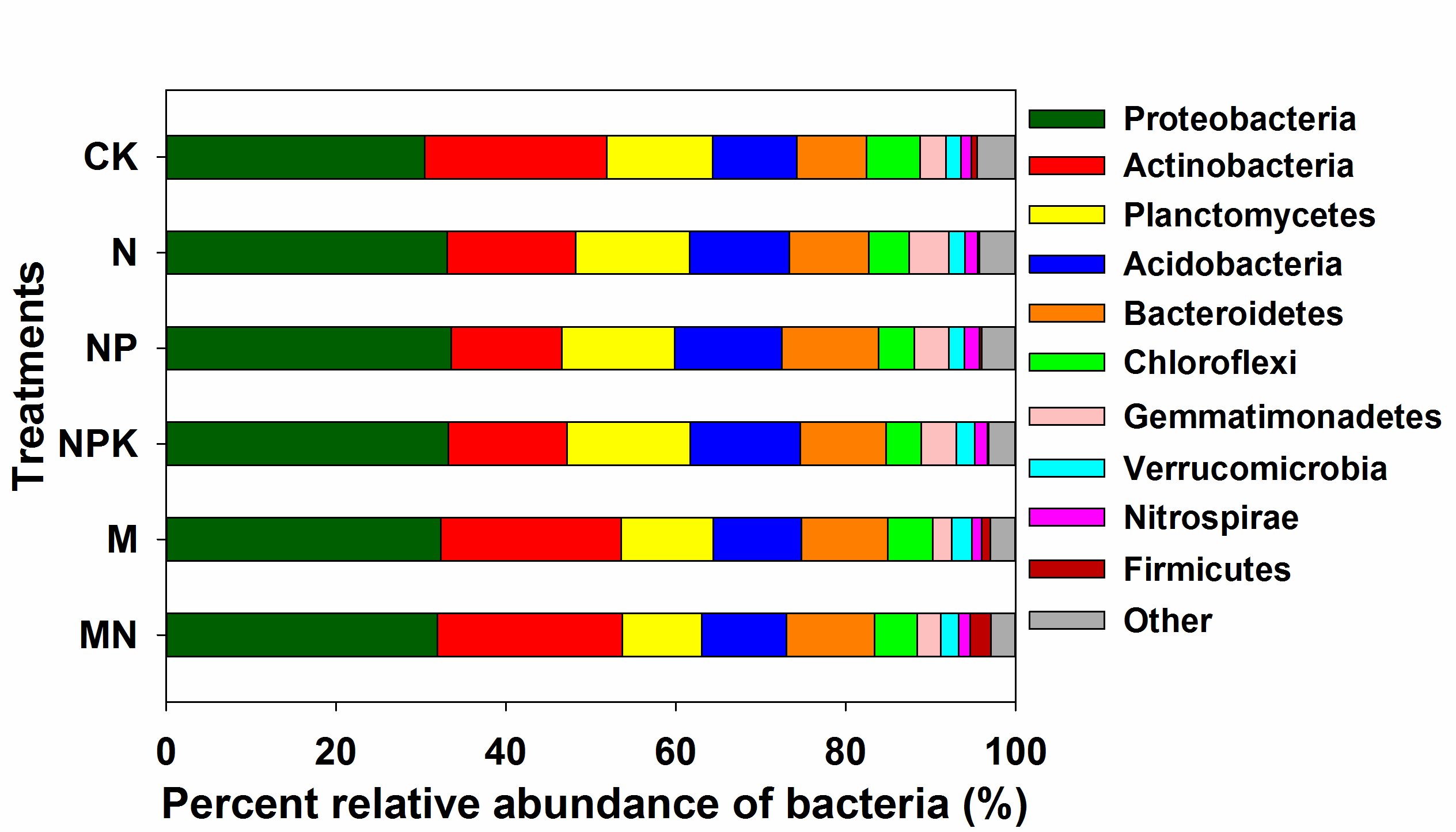


**Figure S11.** Average percentages of total 16S rRNA sequences classified to each phylum among different treatments. Phyla are displayed if they represent at least 1% of the total sequences in at least one treatment. Others contain the taxa with maximum abundance of < 1% in any sample.


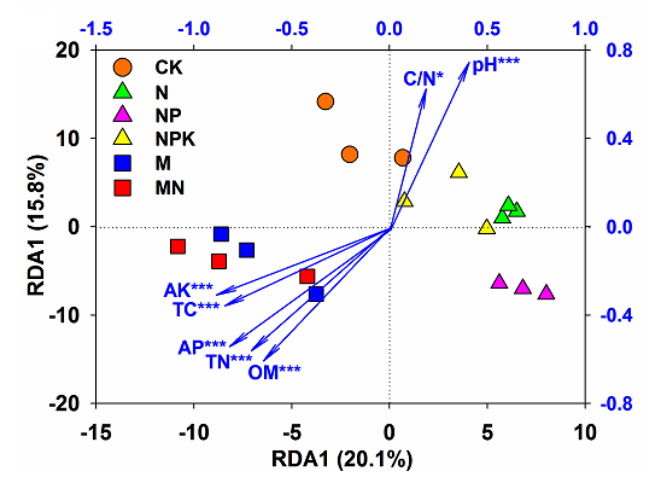


**Figure S12.** Redundancy analysis (RDA) assessed the contributions of soil properties to the structure of the soil bacterial community. Soil properties including pH, C/N, TC, TN, OM, AK, and AP are used in the analysis. The variance inflation factor (vif) is used to check for collinearity between parameters in the two analyses. Parameters showing a significant (*P* < 0.05) impact on community structure are shown in the plots.

**Table S1** Soil properties in each fertilization treatment. (Mean ± SD value)

| Samples | pH | OM g/kg | TC g/kg | TN g/kg | C/N | AK mg/kg | AP mg/kg |
| --- | --- | --- | --- | --- | --- | --- | --- |
| CK | 8.12±0.06 a | 21.26±1.82 c | 14.90±0.10 b | 0.87±0.06 d | 17.25±1.20 a | 111.47±3.20 bc | 4.42±2.28 c |
| N | 7.94±0.02 b | 22.60±0.93 c | 14.67±0.06 b | 0.97±0.06 cd | 15.21±0.88 ab | 109.57±0.87 c | 4.42±0.94 c |
| NP | 7.83±0.16 b | 24.29±1.53 bc | 15.17±0.31 b | 0.97±0.06 cd | 15.73±0.96 ab | 108.66±4.05 c | 29.77±3.23 b |
| NPK | 7.89±0.07 b | 21.77±3.97 c | 15.67±0.29 b | 1.03±0.12 c | 15.29±1.69 ab | 118.75±8.48 b | 27.39±5.21 b |
| M | 7.80±0.04 bc | 27.41±1.50 ab | 18.60±2.01 a | 1.20±0.00 b | 15.50±1.67 ab | 137.88±2.56 a | 96.32±13.71 a |
| MN | 7.68±0.02 c | 28.63±2.13 a | 18.27±0.74 a | 1.37±0.06 a | 13.38±0.64 b | 134.73±2.59 a | 97.67±9.55 a |

Note: OM: organic matter; TC: total carbon; TN: total nitrogen; AK: available potassium; AP: available phosphorus.

Different letters indicate significant differences between means in pairwise comparisons (Duncan’s test; *P* < 0.05) for each treatment.

**Table S2** Detailed information about the primers employed in the high-throughput quantitative PCR

| Gene name | Forward Primer | Reverse Primer | Gene type | Mechanism |
| --- | --- | --- | --- | --- |
| 16S rRNA | GGGTTGCGCTCGTTGC | ATGGYTGTCGTCAGCTCGTG | 16S rRNA | NA |
| cIntI-1(class1) | GGCATCCAAGCAGCAAG | AAGCAGACTTGACCTGA | MGEs/Integrase | Integrase |
| intI-1(clinic) | CGAACGAGTGGCGGAGGGTG | TACCCGAGAGCTTGGCACCCA | MGEs/Integrase | Integrase |
| tnpA-01 | CATCATCGGACGGACAGAATT | GTCGGAGATGTGGGTGTAGAAAGT | MGEs/Transposase | Transposase |
| tnpA-02 | GGGCGGGTCGATTGAAA | GTGGGCGGGATCTGCTT | MGEs/Transposase | Transposase |
| tnpA-03 | AATTGATGCGGACGGCTTAA | TCACCAAACTGTTTATGGAGTCGTT | MGEs/Transposase | Transposase |
| tnpA-04 | CCGATCACGGAAAGCTCAAG | GGCTCGCATGACTTCGAATC | MGEs/Transposase | Transposase |
| tnpA-05 | GCCGCACTGTCGATTTTTATC | GCGGGATCTGCCACTTCTT | MGEs/Transposase | Transposase |
| tnpA-07 | GAAACCGATGCTACAATATCCAATTT | CAGCACCGTTTGCAGTGTAAG | MGEs/Transposase | Transposase |
| IS613  Tp614 | AGGTTCGGACTCAATGCAACA  GGAAATCAACGGCATCCAGTT | TTCAGCACATACCGCCTTGAT  CATCCATGCGCTTTTGTCTCT | MGEs/Transposase  MGEs/Transposase | Transposase  Transposase |
| catA1 | GGGTGAGTTTCACCAGTTTTGATT | CACCTTGTCGCCTTGCGTATA | Chloramphenicol | Antibiotic deactivation |
| catB3 | GCACTCGATGCCTTCCAAAA | AGAGCCGATCCAAACGTCAT | Chloramphenicol | Antibiotic deactivation |
| catB8 | CACTCGACGCCTTCCAAAG | CCGAGCCTATCCAGACATCATT | Chloramphenicol | Antibiotic deactivation |
| cfr | GCAAAATTCAGAGCAAGTTACGAA | AAAATGACTCCCAACCTGCTTTAT | Chloramphenicol | Antibiotic deactivation |
| cmlA1-01 | TAGGAAGCATCGGAACGTTGAT | CAGACCGAGCACGACTGTTG | Chloramphenicol | Efflux pump |
| cmlA1-02 | AGGAAGCATCGGAACGTTGA | ACAGACCGAGCACGACTGTTG | Chloramphenicol | Efflux pump |
| cmx(A) | GCGATCGCCATCCTCTGT | TCGACACGGAGCCTTGGT | Chloramphenicol | Efflux pump |
| floR | ATTGTCTTCACGGTGTCCGTTA | CCGCGATGTCGTCGAACT | Chloramphenicol | Efflux pump |
| qnrA | AGGATTTCTCACGCCAGGATT | CCGCTTTCAATGAAACTGCAA | Fluoroquinolone | Unknown |
| aac | CCCTGCGTTGTGGCTATGT | TTGGCCACGCCAATCC | Aminoglycoside | Antibiotic deactivation |
| aac(6')I1 | GACCGGATTAAGGCCGATG | CTTGCCTTGATATTCAGTTTTTATAACCA | Aminoglycoside | Antibiotic deactivation |
| aac(6')-II | CGACCCGACTCCGAACAA | GCACGAATCCTGCCTTCTCA | Aminoglycoside | Antibiotic deactivation |
| aac(6')-Ib(akaaacA4)-01 | GTTTGAGAGGCAAGGTACCGTAA | GAATGCCTGGCGTGTTTGA | Aminoglycoside | Antibiotic deactivation |
| aac(6')-Ib(akaaacA4)-02 | CGTCGCCGAGCAACTTG | CGGTACCTTGCCTCTCAAACC | Aminoglycoside | Antibiotic deactivation |
| aac(6')-Ib(akaaacA4)-03 | AGAAGCACGCCCGACACTT | GCTCTCCATTCAGCATTGCA | Aminoglycoside | Antibiotic deactivation |
| aac(6')-ly | GCTTTGCGGATGCCTCAAT | GGAGAACAAAAATACCTTCAAGGAAA | Aminoglycoside | Antibiotic deactivation |
| aacA/aphD | AGAGCCTTGGGAAGATGAAGTTT | TTGATCCATACCATAGACTATCTCATCA | Aminoglycoside | Antibiotic deactivation |
| aacC | CGTCACTTATTCGATGCCCTTAC | GTCGGGCGCGGCATA | Aminoglycoside | Antibiotic deactivation |
| aacC1 | GGTCGTGAGTTCGGAGACGTA | GCAAGTTCCCGAGGTAATCG | Aminoglycoside | Antibiotic deactivation |
| aacC2 | ACGGCATTCTCGATTGCTTT | CCGAGCTTCACGTAAGCATTT | Aminoglycoside | Antibiotic deactivation |
| aacC4 | CGGCGTGGGACACGAT | AGGGAACCTTTGCCATCAACT | Aminoglycoside | Antibiotic deactivation |
| aadA-01 | GTTGTGCACGACGACATCATT | GGCTCGAAGATACCTGCAAGAA | Aminoglycoside | Antibiotic deactivation |
| aadA-02 | CGAGATTCTCCGCGCTGTA | GCTGCCATTCTCCAAATTGC | Aminoglycoside | Antibiotic deactivation |
| aadA1 | AGCTAAGCGCGAACTGCAAT | TGGCTCGAAGATACCTGCAA | Aminoglycoside | Antibiotic deactivation |
| aadA-1-01 | AAAAGCCCGAAGAGGAACTTG | CATCTTTCACAAAGATGTTGCTGTCT | Aminoglycoside | Antibiotic deactivation |
| aadA-1-02 | CGGAATTGAAAAAACTGATCGAA | ATACCGGCTGTCCGTCATTT | Aminoglycoside | Antibiotic deactivation |
| aadA2-01 | ACGGCTCCGCAGTGGAT | GGCCACAGTAACCAACAAATCA | Aminoglycoside | Antibiotic deactivation |
| aadA2-02 | CTTGTCGTGCATGACGACATC | TCGAAGATACCCGCAAGAATG | Aminoglycoside | Antibiotic deactivation |
| aadA2-03 | CAATGACATTCTTGCGGGTATC | GACCTACCAAGGCAACGCTATG | Aminoglycoside | Antibiotic deactivation |
| aadA5-01 | ATCACGATCTTGCGATTTTGCT | CTGCGGATGGGCCTAGAAG | Aminoglycoside | Antibiotic deactivation |
| aadA5-02 | GTTCTTGCTCTTGCTCGCATT | GATGCTCGGCAGGCAAAC | Aminoglycoside | Antibiotic deactivation |
| aadA9-01 | CGCGGCAAGCCTATCTTG | CAAATCAGCGACCGCAGACT | Aminoglycoside | Antibiotic deactivation |
| aadA9-02 | GGATGCACGCTTGGATGAA | CCTCTAGCGGCCGGAGTATT | Aminoglycoside | Antibiotic deactivation |
| aadD | CCGACAACATTTCTACCATCCTT | ACCGAAGCGCTCGTCGTATA | Aminoglycoside | Antibiotic deactivation |
| aadE | TACCTTATTGCCCTTGGAAGAGTTA | GGAACTATGTCCCTTTTAATTCTACAATCT | Aminoglycoside | Antibiotic deactivation |
| aph | TTTCAGCAAGTGGATCATGTTAAAAT | CCAAGCTGTTTCCACTGTTTTTC | Aminoglycoside | Antibiotic deactivation |
| aph(2')-Id-01 | TGAGCAGTATCATAAGTTGAGTGAAAAG | GACAGAACAATCAATCTCTATGGAATG | Aminoglycoside | Antibiotic deactivation |
| aph(2')-Id-02 | TAAGGATATACCGACAGTTTTGGAAA | TTTAATCCCTCTTCATACCAATCCATA | Aminoglycoside | Antibiotic deactivation |
| aph6ia | CCCATCCCATGTGTAAGGAAA | GCCACCGCTTCTGCTGTAC | Aminoglycoside | Antibiotic deactivation |
| aphA1(aka kanR) | TGAACAAGTCTGGAAAGAAATGCA | CCTATTAATTTCCCCTCGTCAAAAA | Aminoglycoside | Antibiotic deactivation |
| spcN-01 | AAAAGTTCGATGAAACACGCCTAT | TCCAGTGGTAGTCCCCGAATC | Aminoglycoside | Antibiotic deactivation |
| spcN-02 | CAGAATCTTCCTGAAAAGTTTGATGAA | CGCAGACACGCCGAATC | Aminoglycoside | Antibiotic deactivation |
| str | AATGAGTTTTGGAGTGTCTCAACGTA | AATCAAAACCCCTATTAAAGCCAAT | Aminoglycoside | Antibiotic deactivation |
| strA | CCGGTGGCATTTGAGAAAAA | GTGGCTCAACCTGCGAAAAG | Aminoglycoside | Antibiotic deactivation |
| strB | GCTCGGTCGTGAGAACAATCT | CAATTTCGGTCGCCTGGTAGT | Aminoglycoside | Antibiotic deactivation |
| ampC/blaDHA | TGGCCGCAGCAGAAAGA | CCGTTTTATGCACCCAGGAA | Beta-lactamase | Antibiotic deactivation |
| ampC-01 | TGGCGTATCGGGTCAATGT | CTCCACGGGCCAGTTGAG | Beta-lactam | Antibiotic deactivation |
| ampC-02 | GCAGCACGCCCCGTAA | TGTACCCATGATGCGCGTACT | Beta-lactam | Antibiotic deactivation |
| ampC-04 | TCCGGTGACGCGACAGA | CAGCACGCCGGTGAAAGT | Beta-lactam | Antibiotic deactivation |
| ampC-05 | CTGTTCGAGCTGGGTTCTATAAGTAAA | CAGTATCTGGTCACCGGATCGT | Beta-lactam | Antibiotic deactivation |
| ampC-06 | CCGCTCAAGCTGGACCATAC | CCATATCCTGCACGTTGGTTT | Beta-lactam | Antibiotic deactivation |
| ampC-07 | CCGCCCAGAGCAAGGACTA | GCTCGACTTCACGCCGTAAG | Beta-lactam | Antibiotic deactivation |
| ampC-09 | CAGCCGCTGATGAAAAAATATG | CAGCGAGCCCACTTCGA | Beta-lactam | Antibiotic deactivation |
| bla1 | GCAAGTTGAAGCGAAAGAAAAGA | TACCAGTATCAATCGCATATACACCTAA | Beta-lactam | Antibiotic deactivation |
| bla-AAC-1 | CACACAGCTGATGGCTTATCTAAAA | AATAAACGCGATGGGTTCCA | Beta-lactam | Antibiotic deactivation |
| blaCMY | CCGCGGCGAAATTAAGC | GCCACTGTTTGCCTGTCAGTT | Beta-lactam | Antibiotic deactivation |
| blaCMY2-01 | AAAGCCTCAT GGGTGCATAAA | ATAGCTTTTGTTTGCCAGCATCA | Beta-lactam | Antibiotic deactivation |
| blaCMY2-02 | GCGAGCAGCCTGAAGCA | CGGATGGGCTTGTCCTCTT | Beta-lactam | Antibiotic deactivation |
| blaCTX-M-01 | GGAGGCGTGACGGCTTTT | TTCAGTGCGATCCAGACGAA | Beta-lactam | Antibiotic deactivation |
| blaCTX-M-02 | GCCGCGGTGCTGAAGA | ATCGGATTATAGTTAACCAGGTCAGATTT | Beta-lactam | Antibiotic deactivation |
| blaCTX-M-03 | CGATACCACCACGCCGTTA | GCATTGCCCAACGTCAGATT | Beta-lactam | Antibiotic deactivation |
| blaCTX-M-04 | CTTGGCGTTGCGCTGAT | CGTTCATCGGCACGGTAGA | Beta-lactam | Antibiotic deactivation |
| blaCTX-M-05 | GCGATAACGTGGCGATGAAT | GTCGAGACGGAACGTTTCGT | Beta-lactam | Antibiotic deactivation |
| blaCTX-M-06 | CACAGTTGGTGACGTGGCTTAA | CTCCGCTGCCGGTTTTATC | Beta-lactam | Antibiotic deactivation |
| blaGES | GCAATGTGCTCAACGTTCAAG | GTGCCTGAGTCAATTCTTTCAAAG | Beta-lactam | Antibiotic deactivation |
| blaIMP-01 | AACACGGTTTGGTGGTTCTTGTA | GCGCTCCACAAACCAATTG | Beta-lactam | Antibiotic deactivation |
| blaIMP-02 | AAGGCAGCATTTCCTCTCATTTT | GGATAGATCGAGAATTAAGCCACTCT | Beta-lactam | Antibiotic deactivation |
| bla-L1 | CACCGGGTTACCAGCTGAAG | GCGAAGCTGCGCTTGTAGTC | Beta-lactam | Antibiotic deactivation |
| blaMOX/blaCMY | CTATGTCAATGTGCCGAAGCA | GGCTTGTCCTCTTTCGAATAGC | Beta-lactam | Antibiotic deactivation |
| blaOCH | GGCGACTTGCGCCGTAT | TTTTCTGCTCGGCCATGAG | Beta-lactam | Antibiotic deactivation |
| blaOKP | GCCGCCATCACCATGAG | GGTGACGTTGTCACCGATCTG | Beta-lactam | Antibiotic deactivation |
| blaOXA1/blaOXA30 | CGGATGGTTTGAAGGGTTTATTAT | TCTTGGCTTTTATGCTTGATGTTAA | Beta-lactam | Antibiotic deactivation |
| blaOXA10-01 | CGCAATTATCGGCCTAGAAACT | TTGGCTTTCCGTCCCATTT | Beta-lactam | Antibiotic deactivation |
| blaOXA10-02 | CGCAATTATCGGCCTAGAAACT | TTGGCTTTCCGTCCCATTT | Beta-lactam | Antibiotic deactivation |
| blaOXY | CGTTCAGGCGGCAGGTT | GCCGCGATATAAGATTTGAGAATT | Beta-lactam | Antibiotic deactivation |
| blaPAO | CGCCGTACAACCGGTGAT | GAAGTAATGCGGTTCTCCTTTCA | Beta-lactam | Antibiotic deactivation |
| blaPER | TGCTGGTTGCTGTTTTTGTGA | CCTGCGCAATGATAGCTTCAT | Beta-lactam | Antibiotic deactivation |
| blaPSE | TTGTGACCTATTCCCCTGTAATAGAA | TGCGAAGCACGCATCATC | Beta-lactam | Antibiotic deactivation |
| blaROB | GCAAAGGCATGACGATTGC | CGCGCTGTTGTCGCTAAA | Beta-lactam | Antibiotic deactivation |
| blaSFO | CCGCCGCCATCCAGTA | GGGCCGCCAAGATGCT | Beta-lactam | Antibiotic deactivation |
| blaSHV-01 | TCCCATGATGAGCACCTTTAAA | TTCGTCACCGGCATCCA | Beta-lactam | Antibiotic deactivation |
| blaSHV-02 | CTTTCCCATGATGAGCACCTTT | TCCTGCTGGCGATAGTGGAT | Beta-lactam | Antibiotic deactivation |
| blaTEM | AGCATCTTACGGATGGCATGA | TCCTCCGATCGTTGTCAGAAGT | Beta-lactam | Antibiotic deactivation |
| blaTLA | ACACTTTGCCATTGCTGTTTATGT | TGCAAATTTCGGCAATAATCTTT | Beta-lactam | Antibiotic deactivation |
| blaVEB | CCCGATGCAAAGCGTTATG | GAAAGATTCCCTTTATCTATCTCAGACAA | Beta-lactam | Antibiotic deactivation |
| blaVIM | GCACTTCTCGCGGAGATTG | CGACGGTGATGCGTACGTT | Beta-lactam | Antibiotic deactivation |
| blaZ | GGAGATAAAGTAACAAATCCAGTTAGATATGA | TGCTTAATTTTCCATTTGCGATAAG | Beta-lactam | Antibiotic deactivation |
| cepA | AGTTGCGCAGAACAGTCCTCTT | TCGTATCTTGCCCGTCGATAAT | Beta-lactam | Antibiotic deactivation |
| cfiA | GCAGCGTTGCTGGACACA | GTTCGGGATAAACGTGGTGACT | Beta-lactam | Antibiotic deactivation |
| cfxA | TCATTCCTCGTTCAAGTTTTCAGA | TGCAGCACCAAGAGGAGATGT | Beta-lactam | Antibiotic deactivation |
| cphA-01 | GCGAGCTGCACAAGCTGAT | CGGCCCAGTCGCTCTTC | Beta-lactam | Antibiotic deactivation |
| cphA-02 | GTGCTGATGGCGAGTTTCTG | GGTGTGGTAGTTGGTGTTGATCAC | Beta-lactam | Antibiotic deactivation |
| fox5 | GGTTTGCCGCTGCAGTTC | GCGGCCAGGTGACCAA | Beta-lactam | Antibiotic deactivation |
| mecA | GGTTACGGACAAGGTGAAATACTGAT | TGTCTTTTAATAAGTGAGGTGCGTTAATA | Beta-lactam | Cellular protection |
| ndm-1 | ATTAGCCGCTGCATTGAT | CATGTCGAGATAGGAAGTG | Beta-lactam | Antibiotic deactivation |
| pbp | CCGGTGCCATTGGTTTAGA | AAAATAGCCGCCCCAAGATT | Beta-lactam | Cellular protection |
| pbp2x | TTTCATAAGTATCTGGACATGGAAGAA | CCAAAGGAAACTTGCTTGAGATTAG | Beta-lactam | Cellular protection |
| Pbp5 | GGCGAACTTCTAATTAATCCTATCCA | CGCCGATGACATTCTTCTTATCTT | Beta-lactam | Cellular protection |
| penA | AGACGGTAACGTATAACTTTTTGAAAGA | GCGTGTAGCCGGCAATG | Beta-lactam | Cellular protection |
| carB | GGAGTGAGGCTGACCGTAGAAG | ATCGGCGAAACGCACAAA | MLSB | Efflux pump |
| ereA | CCTGTGGTACGGAGAATTCATGT | ACCGCATTCGCTTTGCTT | MLSB | Antibiotic deactivation |
| ereB | GCTTTATTTCAGGAGGCGGAAT | TTTTAAATGCCACAGCACAGAATC | MLSB | Antibiotic deactivation |
| erm(34) | GCGCGTTGACGACGATTT | TGGTCATACTCGACGGCTAGAAC | MLSB | Cellular protection |
| erm(35) | TTGAAAACGATGTTGCATTAAGTCA | TCTATAATCACAACTAACCACTTGAACGT | MLSB | Cellular protection |
| erm(36) | GGCGGACCGACTTGCAT | TCTGCGTTGACGACGGTTAC | MLSB | Cellular protection |
| ermA | TTGAGAAGGGATTTGCGAAAAG | ATATCCATCTCCACCATTAATAGTAAACC | MLSB | Cellular protection |
| ermA/ermTR | ACATTTTACCAAGGAACTTGTGGAA | GTGGCATGACATAAACCTTCATCA | MLSB | Cellular protection |
| ermB | TAAAGGGCATTTAACGACGAAACT | TTTATACCTCTGTTTGTTAGGGAATTGAA | MLSB | Cellular protection |
| ermC | TTTGAAATCGGCTCAGGAAAA | ATGGTCTATTTCAATGGCAGTTACG | MLSB | Cellular protection |
| ermF | CAGCTTTGGTTGAACATTTACGAA | AAATTCCTAAAATCACAACCGACAA | MLSB | Cellular protection |
| ermJ/ermD | GGACTCGGCAATGGTCAGAA | CCCCGAAACGCAATATAATGTT | MLSB | Cellular protection |
| ermK-01 | GTTTGATATTGGCATTGTCAGAGAAA | ACCATTGCCGAGTCCACTTT | MLSB | Cellular protection |
| ermK-02 | GAGCCGCAAGCCCCTTT | GTGTTTCATTTGACGCGGAGTAA | MLSB | Cellular protection |
| ermT-01 | GTTCACTAGCACTATTTTTAATGACAGAAGT | GAAGGGTGTCTTTTTAATACAATTAACGA | MLSB | Cellular protection |
| ermT-02 | GTAAAATCCCTAGAGAATACTTTCATCCA | TGAGTGATATTTTTGAAGGGTGTCTT | MLSB | Cellular protection |
| ermX | GCTCAGTGGTCCCCATGGT | ATCCCCCCGTCAACGTTT | MLSB | Cellular protection |
| ermY | TTGTCTTTGAAAGTGAAGCAACAGT | TAACGCTAGAGAACGATTTGTATTGAG | MLSB | Cellular protection |
| lmrA-01 | TCGACGTGACCGTAGTGAACA | CGTGACTACCCAGGTGAGTTGA | MLSB | Efflux pump |
| lnuA-01 | TGACGCTCAACACACTCAAAAA | TTCATGCTTAAGTTCCATACGTGAA | MLSB | Antibiotic deactivation |
| lnuB-01 | TGAACATAATCCCCTCGTTTAAAGAT | TAATTGCCCTGTTTCATCGTAAATAA | MLSB | Antibiotic deactivation |
| lnuB-02 | AAAGGAGAAGGTGACCAATACTCTGA | GGAGCTACGTCAAACAACCAGTT | MLSB | Antibiotic deactivation |
| lnuC | TGGTCAATATAACAGATGTAAACCAGATTT | CACCCCAGCCACCATCAA | MLSB | Antibiotic deactivation |
| matA/mel | TAGTAGGCAAGCTCGGTGTTGA | CCTGTGCTATTTTAAGCCTTGTTTCT | MLSB | Efflux pump |
| mdtA | CCTAACGGGCGTGACTTCA | TTCACCTGTTTCAAGGGTCAAA | MLSB | Efflux pump |
| mefA | CCGTAGCATTGGAACAGCTTTT | AAACGGAGTATAAGAGTGCTGCAA | MLSB | Efflux pump |
| mphA-01 | CTGACGCGCTCCGTGTT | GGTGGTGCATGGCGATCT | MLSB | Antibiotic deactivation |
| mphA-02 | TGATGACCCTGCCATCGA | TTCGCGAGCCCCTCTTC | MLSB | Antibiotic deactivation |
| mphB | CGCAGCGCTTGATCTTGTAG | TTACTGCATCCATACGCTGCTT | MLSB | Antibiotic deactivation |
| mphC | CGTTTGAAGTACCGAATTGGAAA | GCTGCGGGTTTGCCTGTA | MLSB | Antibiotic deactivation |
| msrA-01 | CTGCTAACACAAGTACGATTCCAAAT | TCAAGTAAAGTTGTCTTACCTACACCATT | MLSB | Efflux pump |
| msrC-01 | TCAGACCGGATCGGTTGTC | CCTATTTTTTGGAGTCTTCTCTCTAATGTT | MLSB | Efflux pump |
| oleC | CCCGGAGTCGATGTTCGA | GCCGAAGACGTACACGAACAG | MLSB | Efflux pump |
| pikR1 | TCGACATGCGTGACGAGATT | CCGCGAATTAGGCCAGAA | MLSB | Cellular protection |
| pikR2 | TCGTGGGCCAGGTGAAGA | TTCCCCTTGCCGGTGAA | MLSB | Cellular protection |
| vatB-01 | GGAAAAAGCAACTCCATCTCTTGA | TCCTGGCATAACAGTAACATTCTGA | MLSB | Antibiotic deactivation |
| vatB-02 | TTGGGAAAAAGCAACTCCATCT | CAATCCACACATCATTTCCAACA | MLSB | Antibiotic deactivation |
| vatC-01 | CGGAAATTGGGAACGATGTT | GCAATAATAGCCCCGTTTCCTA | MLSB | Antibiotic deactivation |
| vatC-02 | CGATGTTTGGATTGGACGAGAT | GCTGCAATAATAGCCCCGTTT | MLSB | Antibiotic deactivation |
| vatE-01 | GGTGCCATTATCGGAGCAAAT | TTGGATTGCCACCGACAAT | MLSB | Antibiotic deactivation |
| vatE-02 | GACCGTCCTACCAGGCGTAA | TTGGATTGCCACCGACAATT | MLSB | Antibiotic deactivation |
| vgaA-01 | CGAGTATTGTGGAAAGCAGCTAGTT | CCCGTACCGTTAGAGCCGATA | MLSB | Efflux pump |
| vgaA-02 | GACGGGTATTGTGGAAAGCAA | TTTCCTGTACCATTAGATCCGATAATT | MLSB | Efflux pump |
| vgb-01 | AGGGAGGGTATCCATGCAGAT | ACCAAATGCGCCCGTTT | MLSB | Antibiotic deactivation |
| vgbB-01 | CAGCCGGATTCTGGTCCTT | TACGATCTCCATTCAATTGGGTAAA | MLSB | Efflux pump |
| vgbB-02 | ATACGAGCTGCCTAATAAAGGATCTT | TGTGAACCACAGGGCATTATCA | MLSB | Antibiotic deactivation |
| acrA-01 | CAACGATCGGACGGGTTTC | TGGCGATGCCACCGTACT | Multidrug | Efflux pump |
| acrA-02 | GGTCTATCACCCTACGCGCTATC | GCGCGCACGAACATACC | Multidrug | Efflux pump |
| acrA-03 | CAGACCCGCATCGCATATT | CGACAATTTCGCGCTCATG | Multidrug | Efflux pump |
| acrA-04 | TACTTTGCGCGCCATCTTC | CGTGCGCGAACGAACAT | Multidrug | Efflux pump |
| acrA-05 | CGTGCGCGAACGAACA | ACTTTGCGCGCCATCTTC | Multidrug | Efflux pump |
| acrB-01 | AGTCGGTGTTCGCCGTTAAC | CAAGGAAACGAACGCAATACC | Multidrug | Efflux pump |
| acrF | GCGGCCAGGCACAAAA | TACGCTCTTCCCACGGTTTC | Multidrug | Efflux pump |
| acrR-01 | GCGCTGGAGACACGACAAC | GCCTTGCTGCGAGAACAAA | Multidrug | Efflux pump |
| acrR-02 | GATGATACCCCCTGCTGTGAGA | ACCAAACAAGAAGCGCAAGAA | Multidrug | Efflux pump |
| adeA | CAGTTCGAGCGCCTATTTCTG | CGCCCTGACCGACCAAT | Multidrug | Efflux pump |
| ceoA | ATCAACACGGACCAGGACAAG | GGAAAGTCCGCTCACGATGA | Multidrug | Efflux pump |
| cmeA | GCAGCAAAGAAGAAGCACCAA | AGCAGGGTAAGTAAAACTAAGTGGTAAATCT | Multidrug | Efflux pump |
| cmr | CGGCATCGTCAGTGGAATT | CGGTTCCGAAAAAGATGGAA | Multidrug | Efflux pump |
| emrD | CTCAGCAGTATGGTGGTAAGCATT | ACCAGGCGCCGAAGAAC | Multidrug | Efflux pump |
| marR-01 | GCGGCGTACTGGTGAAGCTA | TGCCCTGGTCGTTGATGA | Multidrug | Efflux pump |
| mdetI1 | ATACAGCAGTGGATATTGGTTTAATTGT | TGCATAAGGTGAATGTTCCATGA | Multidrug | Efflux pump |
| mdtE/yhiU | CGTCGGCGCACTCGTT | TCCAGACGTTGTACGGTAACCA | Multidrug | Efflux pump |
| mepA | ATCGGTCGCTCTTCGTTCAC | ATAAATAGGATCGAGCTGCTGGAT | Multidrug | Efflux pump |
| mexA | AGGACAACGCTATGCAACGAA | CCGGAAAGGGCCGAAAT | Multidrug | Efflux pump |
| mexD | TTGCCACTGGCTTTCATGAG | CACTGCGGAGAACTGTCTGTAGA | Multidrug | Efflux pump |
| mexE | GGTCAGCACCGACAAGGTCTAC | AGCTCGACGTACTTGAGGAACAC | Multidrug | Efflux pump |
| mexF | CCGCGAGAAGGCCAAGA | TTGAGTTCGGCGGTGATGA | Multidrug | Efflux pump |
| mtrC-01 | GGACGGGAAGATGGTCCAA | CGTAGCGTTCCGGTTCGAT | Multidrug | Efflux pump |
| mtrC-02 | CGGAGTCCATCGACCATTTG | ATCGTCGGCAAGGAGAATCA | Multidrug | Efflux pump |
| mtrD-02 | GGTCGGCACGCTCTTGTC | TGAAGAATTTGCGCACCACTAC | Multidrug | Efflux pump |
| mtrD-03 | CCGCCAAGCCGATATAGACA | GGCCGGGTTGCCAAA | Multidrug | Efflux pump |
| oprD | ATGAAGTGGAGCGCCATTG | GGCCACGGCGAACTGA | Multidrug | Efflux pump |
| oprJ | ACGAGAGTGGCGTCGACAA | AAGGCGATCTCGTTGAGGAA | Multidrug | Efflux pump |
| pmrA | TTTGCAGGTTTTGTTCCTAATGC | GCAGAGCCTGATTTCTCCTTTG | Multidrug | Efflux pump |
| putitive multidrug | AATTTTGCCGATTATTGCTGAAA | GATTGTCATCATTCGTTTATCACCAA | Multidrug | Efflux pump |
| qac | CAATAATAACCGAAATAATAGGGACAAGTT | AATAAGTGTTCCTAGTGTTGGCCATAG | Multidrug | Efflux pump |
| qacA | TGGCAATAGGAGCTATGGTGTTT | AAGGTAACACTATTTTCGGTCCAAATC | Multidrug | Efflux pump |
| qacA/qacB | TTTAGGCAGCCTCGCTTCA | CCGAATCCAAATAAAACCCAATAA | Multidrug | Efflux pump |
| qacE*Δ*1-01 | TCGCAACATCCGCATTAAAA | ATGGATTTCAGAACCAGAGAAAGAAA | Multidrug | Efflux pump |
| qacE*Δ*1-02 | CCCCTTCCGCCGTTGT | CGACCAGACTGCATAAGCAACA | Multidrug | Efflux pump |
| qacH-01 | GTGGCAGCTATCGCTTGGAT | CCAACGAACGCCCACAA | Multidrug | Efflux pump |
| qacH-02 | CATCGTGCTTGTGGCAGCTA | TGAACGCCCAGAAGTCTAGTTTT | Multidrug | Efflux pump |
| rarD-02 | TGACGCATCGCGTGATCT | AAATTTTCTGTGGCGTCTGAATC | Multidrug | Efflux pump |
| sdeB | CACTACCGCTTCCGCACTTAA | TGAAAAAACGGGAAAAGTCCAT | Multidrug | Efflux pump |
| tolC-01 | GGCCGAGAACCTGATGCA | AGACTTACGCAATTCCGGGTTA | Multidrug | Efflux pump |
| tolC-02 | CAGGCAGAGAACCTGATGCA | CGCAATTCCGGGTTGCT | Multidrug | Efflux pump |
| tolC-03 | GCCAGGCAGAGAACCTGATG | CGCAATTCCGGGTTGCT | Multidrug | Efflux pump |
| ttgA | ACGCCAATGCCAAACGATT | GTCACGGCGCAGCTTGA | Multidrug | Efflux pump |
| ttgB | TCGCCCTGGATGTACACCTT | ACCATTGCCGACATCAACAAC | Multidrug | Efflux pump |
| yceE/mdtG-01 | TGGCACAAAATATCTGGCAGTT | TTGTGTGGCGATAAGAGCATTAG | Multidrug | Efflux pump |
| yceE/mdtG-02 | TTATCTGTTTTCTGCTCACCTTCTTTT | GCGTGGTGACAAACAGGCTTA | Multidrug | Efflux pump |
| yceL/mdtH-01 | TCGGGATGGTGGGCAAT | CGATAACCGAGCCGATGTAGA | Multidrug | Efflux pump |
| yceL/mdtH-02 | CGCGTGAAACCTTAAGTGCTT | AGACGGCTAAACCCCATATAGCT | Multidrug | Efflux pump |
| yceL/mdtH-03 | CTGCCGTTAAATGGATGTATGC | ACTCCAGCGGGCGATAGG | Multidrug | Efflux pump |
| yidY/mdtL-01 | GCAGTTGCATATCGCCTTCTC | CTTCCCGGCAAACAGCAT | Multidrug | Efflux pump |
| yidY/mdtL-02 | TGCTGATCGGGATTCTGATTG | CAGGCGCGACGAACATAAT | Multidrug | Efflux pump |
| fabK | TTTCAGCTCAGCACTTTGGTCAT | AAGGCATCTTTTTCAGCCAGTTC | Other | Antibiotic deactivation |
| imiR | CCGGACTAGAGCTTCATGTAAGC | CCCACGCGGTACTCTTGTAAA | Other | Unknown |
| nisB | GGGAGAGTTGCCGATGTTGTA | AGCCACTCGTTAAAGGGCAAT | Other | Unknown |
| speA | GCAAGAGGTATTTGCTCAACAAGA | CAGGGTCACCCTCATAAAGAAAA | Other | Unknown |
| bacA-01 | CGGCTTCGTGACCTCGTT | ACAATGCGATACCAGGCAAAT | Other/bacitracin | Antibiotic deactivation |
| bacA-02 | TTCCACGACACGATTAAGTCATTG | CGGCTCTTTCGGCTTCAG | Other/bacitracin | Antibiotic deactivation |
| fosB | TCACTGTAACTAATGAAGCATTAGACCAT | CCATCTGGATCTGTAAAGTAAAGAGATC | Other/fosfomycin | Antibiotic deactivation |
| fosX | GATTAAGCCATATCACTTTAATTGTGAAAG | TCTCCTTCCATAATGCAAATCCA | Other/fosfomycin | Antibiotic deactivation |
| nimE | TGCGCCAAGATAGGGCATA | GTCGTGAATTCGGCAGGTTTA | Other/nitroimidazole | Unknown |
| pncA | GCAATCGAGGCGGTGTTC | TTGCCGCAGCCAATTCA | Other/Pyrazinamide | Unknown |
| sat4 | GAATGGGCAAAGCATAAAAACTTG | CCGATTTTGAAACCACAATTATGATA | Other/streptothricin | Antibiotic deactivation |
| dfrA1 | GGAATGGCCCTGATATTCCA | AGTCTTGCGTCCAACCAACAG | Sulfonamide | Antibiotic deactivation |
| dfrA12 | CCTCTACCGAACCGTCACACA | GCGACAGCGTTGAAACAACTAC | Sulfonamide | Antibiotic deactivation |
| folA | CGAGCAGTTCCTGCCAAAG | CCCAGTCATCCGGTTCATAATC | Sulfonamide | Antibiotic deactivation |
| sul1 | CAGCGCTATGCGCTCAAG | ATCCCGCTGCGCTGAGT | Sulfonamide | Cellular protection |
| sul2 | TCATCTGCCAAACTCGTCGTTA | GTCAAAGAACGCCGCAATGT | Sulfonamide | Cellular protection |
| sulA/folP-01 | CAGGCTCGTAAATTGATAGCAGAAG | CTTTCCTTGCGAATCGCTTT | Sulfonamide | Cellular protection |
| sulA/folP-03 | CACGGCTTCGGCTCATGT | TGCCATCCTGTGACTAGCTACGT | Sulfonamide | Cellular protection |
| tet(32) | CCATTACTTCGGACAACGGTAGA | CAATCTCTGTGAGGGCATTTAACA | Tetracycline | Cellular protection |
| tet(34) | CTTAGCGCAAACAGCAATCAGT | CGGTGATACAGCGCGTAAACT | Tetracycline | Unknown |
| tet(35) | ACCCCATGACGTACCTGTAGAGA | CAACCCACACTGGCTACCAGTT | Tetracycline | Unknown |
| tet(36)-01 | AGAATACTCAGCAGAGGTCAGTTCCT | TGGTAGGTCGATAACCCGAAAAT | Tetracycline | Cellular protection |
| tet(36)-02 | TGCAGGAAAGACCTCCATTACAG | CTTTGTCCACACTTCCACGTACTATG | Tetracycline | Cellular protection |
| tet(37) | GAGAACGTTGAAAAGGTGGTGAA | AACCAAGCCTGGATCAGTCTCA | Tetracycline | Unknown |
| tetA-01 | GCTGTTTGTTCTGCCGGAAA | GGTTAAGTTCCTTGAACGCAAACT | Tetracycline | Efflux pump |
| tetA-02 | CTCACCAGCCTGACCTCGAT | CACGTTGTTATAGAAGCCGCATAG | Tetracycline | Efflux pump |
| tetB-01 | AGTGCGCTTTGGATGCTGTA | AGCCCCAGTAGCTCCTGTGA | Tetracycline | Efflux pump |
| tetB-02 | GCCCAGTGCTGTTGTTGTCAT | TGAAAGCAAACGGCCTAAATACA | Tetracycline | Efflux pump |
| tetC-01 | CATATCGCAATACATGCGAAAAA | AAAGCCGCGGTAAATAGCAA | Tetracycline | Efflux pump |
| tetC-02 | ACTGGTAAGGTAAACGCCATTGTC | ATGCATAAACCAGCCATTGAGTAAG | Tetracycline | Efflux pump |
| tetD-01 | TGCCGCGTTTGATTACACA | CACCAGTGATCCCGGAGATAA | Tetracycline | Efflux pump |
| tetD-02 | TGTCATCGCGCTGGTGATT | CATCCGCTTCCGGGAGAT | Tetracycline | Efflux pump |
| tetE | TTGGCGCTGTATGCAATGAT | CGACGACCTATGCGATCTGA | Tetracycline | Efflux pump |
| tetG-01 | TCAACCATTGCCGATTCGA | TGGCCCGGCAATCATG | Tetracycline | Efflux pump |
| tetG-02 | CATCAGCGCCGGTCTTATG | CCCCATGTAGCCGAACCA | Tetracycline | Efflux pump |
| tetH | TTTGGGTCATCTTACCAGCATTAA | TTGCGCATTATCATCGACAGA | Tetracycline | Efflux pump |
| tetJ | GGGTGCCGCATTAGATTACCT | TCGTCCAATGTAGAGCATCCATA | Tetracycline | Efflux pump |
| tetK | CAGCAGTCATTGGAAAATTATCTGATTATA | CCTTGTACTAACCTACCAAAAATCAAAATA | Tetracycline | Efflux pump |
| tetL-01 | AGCCCGATTTATTCAAGGAATTG | CAAATGCTTTCCCCCTGTTCT | Tetracycline | Efflux pump |
| tetL-02 | ATGGTTGTAGTTGCGCGCTATAT | ATCGCTGGACCGACTCCTT | Tetracycline | Efflux pump |
| tetM-01 | CATCATAGACACGCCAGGACATAT | CGCCATCTTTTGCAGAAATCA | Tetracycline | Cellular protection |
| tetM-02 | TAATATTGGAGTTTTAGCTCATGTTGATG | CCTCTCTGACGTTCTAAAAGCGTATTAT | Tetracycline | Cellular protection |
| tetO-01 | ATGTGGATACTACAACGCATGAGATT | TGCCTCCACATGATATTTTTCCT | Tetracycline | Cellular protection |
| tetPA | AGTTGCAGATGTGTATAGTCGTAAACTATCTATT | TGCTACAAGTACGAAAACAAAACTAGAA | Tetracycline | Efflux pump |
| tetPB-01 | ACACCTGGACACGCTGATTTT | ACCGTCTAGAACGCGGAATG | Tetracycline | Cellular protection |
| tetPB-02 | TGATACACCTGGACACGCTGAT | CGTCCAAAACGCGGAATG | Tetracycline | Cellular protection |
| tetPB-03 | TGGGCGACAGTAGGCTTAGAA | TGACCCTACTGAAACATTAGAAATATACCT | Tetracycline | Cellular protection |
| tetPB-04 | AGTGGTGCAAATACTGAAAAAGTTGT | TTTGTTCCTTCGTTTTGGACAGA | Tetracycline | Cellular protection |
| tetPB-05 | CTGAAGTGGAGCGATCATTCC | CCCTCAACGGCAGAAATAACTAA | Tetracycline | Cellular protection |
| tetQ | CGCCTCAGAAGTAAGTTCATACACTAAG | TCGTTCATGCGGATATTATCAGAAT | Tetracycline | Cellular protection |
| tetR-02 | CGCGATAGACGCCTTCGA | TCCTGACAACGAGCCTCCTT | Tetracycline | Efflux pump |
| tetR-03 | CGCGATGGAGCAAAAGTACAT | AGTGAAAAACCTTGTTGGCATAAAA | Tetracycline | Efflux pump |
| tetS | TTAAGGACAAACTTTCTGACGACATC | TGTCTCCCATTGTTCTGGTTCA | Tetracycline | Cellular protection |
| tetT | CCATATAGAGGTTCCACCAAATCC | TGACCCTATTGGTAGTGGTTCTATTG | Tetracycline | Cellular protection |
| tetU-01 | GTGGCAAAGCAACGGATTG | TGCGGGCTTGCAAAACTATC | Tetracycline | Unknown |
| tetV | GCGGGAACGACGATGTATATC | CCGCTATCTCACGACCATGAT | Tetracycline | Efflux pump |
| tetX | AAATTTGTTACCGACACGGAAGTT | CATAGCTGAAAAAATCCAGGACAGTT | Tetracycline | Unknown |
| vanA | AAAAGGCTCTGAAAACGCAGTTAT | CGGCCGTTATCTTGTAAAAACAT | Vancomycin | Cellular protection |
| vanB-01 | TTGTCGGCGAAGTGGATCA | AGCCTTTTTCCGGCTCGTT | Vancomycin | Cellular protection |
| vanB-02 | CCGGTCGAGGAACGAAATC | TCCTCCTGCAAAAAAAGATCAAC | Vancomycin | Cellular protection |
| vanC-01 | ACAGGGATTGGCTATGAACCAT | TGACTGGCGATGATTTGACTATG | Vancomycin | Cellular protection |
| vanC-03 | AAATCAATACTATGCCGGGCTTT | CCGACCGCTGCCATCA | Vancomycin | Cellular protection |
| vanC1 | AGGCGATAGCGGGTATTGAA | CAATCGTCAATTGCTCATTTCC | Vancomycin | Cellular protection |
| vanC2/vanC3 | TTTGACTGTCGGTGCTTGTGA | TCAATCGTTTCAGGCAATGG | Vancomycin | Cellular protection |
| vanG | ATTTGAATTGGCAGGTATACAGGTTA | TGATTTGTCTTTGTCCATACATAATGC | Vancomycin | Cellular protection |
| vanHB | GAGGTTTCCGAGGCGACAA | CTCTCGGCGGCAGTCGTAT | Vancomycin | Cellular protection |
| vanHD | GTGGCCGATTATACCGTCATG | CGCAGGTCATTCAGGCAAT | Vancomycin | Cellular protection |
| vanRA-01 | CCCTTACTCCCACCGAGTTTT | TTCGTCGCCCCATATCTCAT | Vancomycin | Cellular protection |
| vanRA-02 | CCACTCCGGCCTTGTCATT | GCTAACCACATTCCCCTTGTTTT | Vancomycin | Cellular protection |
| vanRB | GCCCTGTCGGATGACGAA | TTACATAGTCGTCTGCCTCTGCAT | Vancomycin | Cellular protection |
| vanRC | TGCGGGAAAAACTGAACGA | CCCCCCATACGGTTTTGATTA | Vancomycin | Cellular protection |
| vanRC4 | AGTGCTTTGGCTTATCTCGAAAA | TCCGGCAGCATCACATCTAA | Vancomycin | Cellular protection |
| vanRD | TTATAATGGCAAGGATGCACTAAAGT | CGTCTACATCCGGAAGCATGA | Vancomycin | Cellular protection |
| vanSA | CGCGTCATGCTTTCAAAATTC | TCCGCAGAAAGCTCAATTTGTT | Vancomycin | Cellular protection |
| vanSB | GCGCGGCAAATGACAAC | TTTGCCATTTTATTCGCACTGT | Vancomycin | Cellular protection |
| vanSC-02 | GCCATCAGCGAGTCTGATGA | CAGCTGGGATCGTTTTTCCTT | Vancomycin | Cellular protection |
| vanSE | TGGCCGAAGAAGCAGGAA | CAATAATACTCGTCAAAGGAGTTCTCA | Vancomycin | Cellular protection |
| vanTC-01 | CACACGCATTTTTTCCCATCTAG | CAGCCAACAGATCATCAAAACAA | Vancomycin | Cellular protection |
| vanTC-02 | ACAGTTGCCGCTGGTGAAG | CGTGGCTGGTCGATCAAAA | Vancomycin | Cellular protection |
| vanTE | GTGGTGCCAAGGAAGTTGCT | CGTAGCCACCGCAAAAAAAT | Vancomycin | Cellular protection |
| vanTG | CGTGTAGCCGTTCCGTTCTT | CGGCATTACAGGTATATCTGGAAA | Vancomycin | Cellular protection |
| vanWB | CGGACAAAGATACCCCCTATAAAG | AAATAGTAAATTGCTCATCTGGCACAT | Vancomycin | Cellular protection |
| vanWG | ACATTTTCATTTTGGCAGCTTGTAC | CCGCCATAAGAGCCTACAATCT | Vancomycin | Cellular protection |
| vanXA | CGCTAAATATGCCACTTGGGATA | TCAAAAGCGATTCAGCCAACT | Vancomycin | Cellular protection |
| VanXB | AGGCACAAAATCGAAGATGCTT | GGGTATGGCTCATCAATCAACTT | Vancomycin | Cellular protection |
| VanXD | TAAACCGTGTTATGGGAACGAA | GCGATAGCCGTCCCATAAGA | Vancomycin | Cellular protection |
| VanYB | GGCTAAAGCGGAAGCAGAAA | GATATCCACAGCAAGACCAAGCT | Vancomycin | Cellular protection |
| vanYD-01 | AAGGCGATACCCTGACTGTCA | ATTGCCGGACGGAAGCA | Vancomycin | Cellular protection |
| vanYD-02 | CAAACGGAAGAGAGGTCACTTACA | CGGACGGTAATAGGGACTGTTC | Vancomycin | Cellular protection |

MLSB: macrolide-lincosamide-streptogramin B

1. Stedtfeld, R. D.; Baushke, S. W.; Tourlousse, D. M.; Miller, S. M.; Stedtfeld, T. M.; Gulari, E.; Tiedje, J. M.; Hashsham, S. A. Development and experimental validation of a predictive threshold cycle equation for quantification of virulence and marker genes by high-throughput nanoliter-volume PCR on the openarray platform. *Applied. Environ. Microbiol.* **2011**, *74* (12), 3831-3838.
2. Zhu, Y. G.; Johnson, T. A.; Su, J. Q.; Qiao, M.; Guo, G. X.; Stedtfeld, R. D.; Hashsham, S. A.; Tiedje, J. M. Diverse and abundant antibiotic resistance genes in Chinese swine farms. *PNAS*. **2013**, *110* (9), 3435-3440.

**Table S3** Relative abundance of ARGs in pig manure (PM) and soil samples

| Classification | Mechanism | Gene | CK | N | NP | NPK | M | MN | PM |
| --- | --- | --- | --- | --- | --- | --- | --- | --- | --- |
| Aminoglycoside | deactivate | aac(6')-Ib(aka aacA4) |  |  |  |  | 1.53E-03 | 1.34E-03 | 3.23E-02 |
| Aminoglycoside | deactivate | aac(6')-II |  |  |  | 2.35E-05 | 2.97E-04 | 1.20E-04 | 1.19E-02 |
| Aminoglycoside | deactivate | aacA_aphD |  |  |  |  | 5.21E-05 | 4.15E-05 | 2.20E-02 |
| Aminoglycoside | deactivate | aacC |  | 7.59E-04 | 9.33E-04 | 1.02E-03 |  | 2.71E-03 |  |
| Aminoglycoside | deactivate | aacC1 |  |  |  |  | 1.71E-04 |  |  |
| Aminoglycoside | deactivate | aacC2 |  |  |  |  | 1.84E-04 |  | 4.63E-03 |
| Aminoglycoside | deactivate | aacC4 | 4.35E-05 | 2.32E-04 | 1.70E-04 | 1.30E-04 |  | 5.21E-04 | 4.79E-03 |
| Aminoglycoside | deactivate | aadA | 9.52E-05 | 8.24E-05 | 1.41E-04 | 1.42E-04 | 1.03E-02 | 2.03E-02 | 1.34E-01 |
| Aminoglycoside | deactivate | aadA1 |  | 1.07E-04 | 5.89E-05 | 5.63E-05 | 3.55E-04 | 4.59E-04 | 2.51E-02 |
| Aminoglycoside | deactivate | aadA2 | 9.95E-05 | 1.33E-04 | 2.04E-04 |  | 8.18E-03 | 2.01E-02 | 1.28E-01 |
| Aminoglycoside | deactivate | aadA5 |  | 6.46E-05 | 6.02E-05 |  | 5.44E-04 | 4.61E-04 | 8.13E-03 |
| Aminoglycoside | deactivate | aadA9 | 3.37E-05 |  | 2.60E-05 |  | 5.83E-04 | 1.39E-03 | 5.72E-03 |
| Aminoglycoside | deactivate | aadD |  |  |  |  |  | 6.88E-05 | 9.47E-03 |
| Aminoglycoside | deactivate | aadE | 6.65E-05 | 6.27E-05 | 7.67E-05 | 9.92E-05 | 1.89E-04 | 8.57E-04 | 2.03E-02 |
| Aminoglycoside | deactivate | aph(2')-Id |  |  |  |  |  |  | 2.66E-03 |
| Aminoglycoside | deactivate | aphA1(aka kanR) |  |  |  |  | 3.82E-04 | 8.43E-04 | 7.36E-03 |
| Aminoglycoside | deactivate | spcN |  |  |  | 2.46E-05 |  | 1.22E-04 |  |
| Aminoglycoside | deactivate | str |  |  |  |  | 7.38E-05 | 1.24E-04 | 1.46E-03 |
| Aminoglycoside | deactivate | strB |  | 1.70E-04 |  |  | 2.11E-03 | 3.06E-03 | 3.11E-02 |
| Beta_lactamase | deactivate | ampC |  | 6.09E-04 | 3.07E-04 | 4.64E-04 |  | 8.05E-05 | 8.45E-05 |
| Beta_lactamase | deactivate | bla1 | 8.54E-04 | 2.97E-04 | 9.72E-04 | 8.31E-04 | 7.55E-04 | 5.90E-04 |  |
| Beta_lactamase | deactivate | blaCMY |  |  | 1.43E-04 | 1.44E-04 |  | 2.33E-04 | 8.99E-05 |
| Beta_lactamase | deactivate | blaCTX-M | 2.07E-04 | 8.53E-04 | 1.02E-03 | 6.34E-04 | 2.11E-04 | 4.80E-04 | 8.90E-05 |
| Beta_lactamase | deactivate | bla-L1 |  |  |  |  |  | 6.40E-05 | 1.43E-05 |
| Beta_lactamase | deactivate | blaOCH |  |  |  |  |  | 7.02E-05 |  |
| Beta_lactamase | deactivate | blaOXA1_blaOXA30 |  |  |  |  |  | 1.28E-04 | 1.74E-03 |
| Beta_lactamase | deactivate | blaOXA10 |  |  |  |  | 9.97E-05 | 1.31E-04 | 2.24E-03 |
| Beta_lactamase | deactivate | blaOXY |  | 1.42E-04 | 8.77E-05 | 8.50E-05 |  | 6.93E-04 | 2.82E-04 |
| Beta_lactamase | deactivate | blaPER |  | 6.01E-05 | 5.77E-05 | 4.85E-05 |  | 5.22E-05 |  |
| Beta_lactamase | deactivate | blaPSE |  |  |  |  | 7.39E-05 | 1.31E-04 | 1.27E-03 |
| Beta_lactamase | deactivate | blaSFO | 9.96E-04 | 2.23E-03 | 1.54E-03 | 1.13E-03 | 1.18E-03 |  | 1.19E-03 |
| Beta_lactamase | deactivate | blaSHV |  | 3.90E-05 | 3.38E-05 | 2.01E-05 |  |  | 3.73E-04 |
| Beta_lactamase | deactivate | blaTEM |  | 1.57E-04 |  | 9.88E-05 | 8.33E-05 |  | 1.42E-04 |
| Beta_lactamase | deactivate | blaVIM |  | 5.18E-05 |  | 3.87E-05 |  | 6.98E-05 | 1.30E-05 |
| Beta_lactamase | deactivate | cfxA |  |  |  |  |  |  | 2.49E-04 |
| Beta_lactamase | deactivate | cphA | 1.64E-02 | 1.57E-02 | 1.32E-02 | 1.13E-02 | 2.68E-02 | 9.59E-02 | 2.56E-02 |
| Beta_lactamase | deactivate | fox5 | 1.72E-03 | 8.57E-04 | 2.68E-03 | 4.69E-03 | 3.27E-03 |  |  |
| Beta_lactamase | deactivate | ndm-1 |  |  |  |  |  |  | 2.12E-05 |
| Beta_lactamase | protection | Pbp5 | 3.34E-04 | 2.03E-04 | 2.46E-04 | 1.19E-04 | 2.03E-04 | 1.77E-04 | 9.95E-05 |
| Beta_lactamase | protection | penA |  | 5.30E-05 | 1.54E-04 | 1.43E-04 |  |  | 1.20E-03 |
| FCA | deactivate | catB3 |  |  |  |  | 3.40E-04 | 4.94E-04 | 7.86E-03 |
| FCA | deactivate | catB8 |  |  |  |  |  |  | 8.66E-04 |
| FCA | deactivate | cfr |  |  |  |  |  |  | 1.77E-04 |
| FCA | efflux | cmlA1 |  | 5.36E-04 | 1.81E-04 |  |  |  | 1.24E-02 |
| FCA | efflux | cmx(A) | 4.16E-03 |  |  |  |  | 1.24E-02 | 4.03E-02 |
| FCA | efflux | floR | 4.21E-05 |  |  |  | 2.98E-03 |  | 2.30E-02 |
| MLSB | deactivate | ereA |  |  |  |  | 4.50E-05 | 2.17E-04 | 8.65E-04 |
| MLSB | protection | erm(34) |  |  | 3.66E-05 |  |  |  | 3.14E-05 |
| MLSB | protection | erm(35) |  |  |  |  |  | 8.59E-05 | 8.96E-04 |
| MLSB | protection | ermA |  |  |  |  |  | 6.07E-05 | 6.02E-04 |
| MLSB | protection | ermA_ermTR |  |  |  |  |  |  | 2.58E-05 |
| MLSB | protection | ermB | 1.02E-04 | 1.02E-04 | 8.11E-05 | 5.96E-05 | 2.54E-04 | 2.01E-04 | 4.95E-02 |
| MLSB | protection | ermC |  |  |  |  |  | 7.61E-05 | 3.15E-03 |
| MLSB | protection | ermF |  |  |  |  | 9.42E-04 | 1.54E-03 | 4.18E-02 |
| MLSB | protection | ermK |  |  |  |  |  | 6.29E-05 |  |
| MLSB | protection | ermT |  |  |  |  | 4.92E-05 | 1.96E-04 | 2.02E-03 |
| MLSB | protection | ermX |  |  | 5.40E-05 |  | 1.68E-04 |  | 4.84E-04 |
| MLSB | protection | ermY |  |  |  |  |  |  | 5.92E-05 |
| MLSB | deactivate | lnuA-01 | 2.45E-05 | 4.35E-05 | 3.15E-05 | 4.14E-05 | 1.83E-04 | 2.47E-04 | 5.36E-03 |
| MLSB | deactivate | lnuB | 3.26E-05 |  | 3.00E-05 |  | 5.43E-04 | 8.87E-04 | 1.14E-02 |
| MLSB | efflux | matA_mel |  |  |  |  | 1.32E-04 |  | 5.06E-03 |
| MLSB | efflux | mdtA |  |  |  |  |  |  | 7.14E-04 |
| MLSB | efflux | mefA |  |  |  |  | 9.86E-05 | 2.79E-04 | 4.86E-03 |
| MLSB | deactivate | mphA | 1.57E-03 | 1.73E-03 | 1.15E-03 | 1.79E-03 | 1.72E-03 | 2.66E-03 | 2.43E-03 |
| MLSB | efflux | msrC-01 | 8.14E-05 | 8.81E-05 | 1.08E-04 | 1.17E-04 | 7.80E-05 | 6.00E-05 |  |
| MLSB | efflux | oleC | 9.53E-04 | 1.54E-03 | 1.33E-03 | 1.07E-03 | 1.45E-03 | 2.94E-03 | 5.52E-04 |
| MLSB | protection | pikR1 |  |  |  |  |  | 2.50E-05 |  |
| MLSB | protection | pikR2 | 5.31E-04 | 8.81E-04 |  |  | 7.82E-04 | 8.93E-04 | 7.34E-04 |
| MLSB | deactivate | vatE | 4.38E-05 | 5.53E-04 | 4.95E-04 | 7.10E-04 | 4.39E-04 | 1.28E-03 | 5.99E-03 |
| MLSB | deactivate | vgb |  |  |  |  |  | 3.95E-05 | 3.42E-05 |
| Multidrug | efflux | acrA | 5.59E-04 | 4.16E-03 | 4.03E-03 | 3.01E-03 | 4.89E-04 | 9.18E-04 | 3.24E-03 |
| Multidrug | efflux | acrF |  |  |  |  |  |  | 5.65E-05 |
| Multidrug | efflux | acrR |  |  |  |  |  |  | 2.19E-05 |
| Multidrug | efflux | adeA |  |  |  |  |  |  | 1.10E-05 |
| Multidrug | efflux | ceoA | 2.60E-04 | 1.21E-03 | 6.97E-04 | 8.34E-04 | 1.84E-04 |  | 2.32E-04 |
| Multidrug | efflux | cmr |  |  |  | 5.51E-04 |  |  |  |
| Multidrug | efflux | emrD | 4.32E-05 |  |  | 1.09E-03 |  |  |  |
| Multidrug | efflux | marR-01 | 9.32E-05 | 8.00E-05 | 8.22E-05 | 6.65E-05 | 9.87E-05 |  |  |
| Multidrug | efflux | mdtE_yhiU | 8.57E-05 | 1.70E-04 | 9.43E-05 | 1.12E-04 |  | 3.74E-04 | 2.22E-04 |
| Multidrug | efflux | mepA |  | 9.65E-04 | 5.99E-04 | 5.89E-04 | 2.19E-04 | 9.76E-04 | 6.54E-04 |
| Multidrug | efflux | mexA |  |  |  | 1.95E-05 |  | 1.59E-04 |  |
| Multidrug | efflux | mexE |  | 2.12E-04 | 1.30E-04 | 1.72E-04 |  | 3.47E-04 | 8.61E-05 |
| Multidrug | efflux | mexF |  |  |  |  |  |  | 2.42E-02 |
| Multidrug | efflux | mtrC |  |  |  | 1.44E-04 |  | 6.03E-05 |  |
| Multidrug | efflux | mtrD |  | 3.07E-04 | 1.54E-04 | 1.31E-04 |  | 2.08E-04 | 6.95E-05 |
| Multidrug | efflux | oprD |  | 2.14E-03 | 1.54E-03 | 1.63E-03 | 1.12E-03 | 2.29E-03 | 2.94E-03 |
| Multidrug | efflux | oprJ | 1.59E-02 | 1.01E-02 | 2.44E-02 | 1.78E-02 | 1.75E-02 |  | 5.75E-03 |
| Multidrug | efflux | putitive multidrug | 1.94E-03 | 1.66E-03 | 1.45E-03 | 1.43E-03 | 1.55E-03 | 1.11E-03 |  |
| Multidrug | efflux | qacEdelta1 | 5.21E-05 | 2.61E-04 | 1.43E-04 | 9.65E-05 | 1.26E-02 | 9.12E-03 | 2.06E-01 |
| Multidrug | efflux | qacH |  | 8.86E-05 |  |  | 4.17E-05 | 2.42E-04 | 1.78E-04 |
| Multidrug | efflux | rarD-02 |  |  |  | 2.32E-05 |  |  |  |
| Multidrug | efflux | tolC | 1.03E-04 | 1.77E-04 | 5.87E-05 | 9.57E-05 |  | 4.08E-04 |  |
| Multidrug | efflux | ttgA | 7.31E-05 |  | 1.04E-04 |  | 1.57E-04 | 1.16E-04 | 5.58E-05 |
| Multidrug | efflux | ttgB |  |  | 2.49E-04 |  | 1.49E-04 |  | 2.24E-04 |
| Multidrug | efflux | yidY_mdtL |  |  |  | 5.21E-05 |  |  | 3.84E-04 |
| Sulfonamides | deactivate | dfrA1 |  |  |  |  | 3.17E-04 | 6.46E-04 | 7.67E-03 |
| Sulfonamides | deactivate | dfrA12 |  |  |  |  |  |  | 2.09E-04 |
| Sulfonamides | protection | sul1 |  |  |  |  |  | 9.73E-05 |  |
| Sulfonamides | protection | sul2 |  |  | 1.18E-04 | 8.83E-05 | 4.30E-03 | 7.20E-03 | 6.53E-02 |
| Sulfonamides | protection | sulA-folP |  | 6.21E-05 | 1.13E-04 |  |  | 1.66E-04 |  |
| Tetracycline | protection | tet(32) |  |  |  |  | 3.64E-04 | 2.47E-04 | 1.20E-02 |
| Tetracycline | unknown | tet(34) |  |  |  | 2.83E-05 |  | 7.11E-05 |  |
| Tetracycline | protection | tet(36) |  |  |  |  |  | 1.72E-04 | 6.16E-04 |
| Tetracycline | efflux | tetA | 5.20E-05 | 1.15E-04 | 8.18E-05 |  |  | 1.44E-03 | 2.60E-02 |
| Tetracycline | efflux | tetB |  | 9.50E-05 | 1.22E-04 | 2.02E-04 | 2.03E-04 | 8.35E-04 | 7.42E-04 |
| Tetracycline | efflux | tetC |  |  |  |  |  |  | 2.63E-04 |
| Tetracycline | efflux | tetD |  | 5.85E-05 | 1.23E-04 | 9.36E-05 |  | 1.04E-04 | 2.28E-05 |
| Tetracycline | efflux | tetE |  |  |  |  |  |  | 9.07E-06 |
| Tetracycline | efflux | tetG | 2.96E-04 | 4.50E-04 |  | 2.69E-04 | 1.55E-02 | 1.73E-02 | 1.04E-01 |
| Tetracycline | efflux | tetH |  |  |  |  |  |  | 8.74E-05 |
| Tetracycline | efflux | tetK |  |  |  |  |  |  | 7.16E-05 |
| Tetracycline | efflux | tetL |  |  |  |  | 2.73E-04 |  | 1.41E-02 |
| Tetracycline | protection | tetM | 7.35E-05 | 4.54E-05 | 6.99E-05 | 2.41E-05 | 1.18E-03 | 2.83E-03 | 5.63E-02 |
| Tetracycline | protection | tetO |  | 3.70E-05 | 2.80E-05 |  |  | 3.96E-04 | 4.33E-03 |
| Tetracycline | efflux | tetPA |  |  |  |  | 1.43E-04 | 3.81E-04 | 8.47E-04 |
| Tetracycline | protection | tetPB | 2.13E-03 | 1.74E-03 | 1.36E-03 | 1.76E-03 | 1.69E-03 | 1.52E-03 | 1.55E-03 |
| Tetracycline | protection | tetQ |  |  |  |  | 2.19E-04 | 2.10E-04 | 9.64E-03 |
| Tetracycline | efflux | tetR | 1.50E-04 |  |  | 1.56E-04 |  | 3.67E-03 | 2.11E-02 |
| Tetracycline | protection | TetS | 7.71E-05 | 1.36E-04 | 9.25E-05 | 6.95E-05 | 6.47E-05 | 7.09E-05 | 1.81E-03 |
| Tetracycline | protection | tetT |  | 2.72E-05 |  |  | 1.75E-04 | 4.23E-04 | 8.03E-04 |
| Tetracycline | unknown | tetX |  | 2.82E-04 |  |  | 1.68E-03 | 2.93E-03 | 1.74E-02 |
| Vancomycin | protection | vanB |  | 5.25E-04 | 2.82E-04 | 4.04E-04 |  | 5.28E-04 | 1.66E-04 |
| Vancomycin | protection | vanC |  | 8.19E-04 |  | 9.03E-04 | 1.24E-03 | 1.99E-03 | 1.22E-03 |
| Vancomycin | protection | vanHB |  |  | 1.88E-04 | 2.89E-04 | 2.33E-04 | 5.64E-04 | 1.93E-04 |
| Vancomycin | protection | vanRA | 1.11E-04 |  | 1.11E-04 | 7.29E-05 | 9.41E-05 | 7.09E-05 |  |
| Vancomycin | protection | vanSB |  |  | 7.20E-05 | 7.88E-05 | 6.53E-05 | 1.09E-04 |  |
| Vancomycin | protection | vanTC |  |  | 6.61E-05 | 1.24E-04 |  |  | 3.33E-05 |
| Vancomycin | protection | vanXD |  |  |  |  |  |  | 7.29E-06 |
| Vancomycin | protection | vanYD | 2.11E-04 |  |  |  |  | 5.55E-04 | 2.59E-04 |
| other/bacitracin | deactivate | bacA |  |  |  |  |  | 9.06E-05 |  |
| other/Pyrazinamide | unknown | pncA | 1.31E-03 | 2.51E-03 | 1.15E-03 | 1.08E-03 | 1.73E-03 | 4.70E-03 | 4.28E-03 |
| other/streptothricin | deactivate | sat4 | 4.95E-05 | 8.49E-05 | 4.42E-05 | 3.06E-05 | 1.67E-04 | 2.57E-04 | 7.52E-03 |
| MGEs | integrase | intI-1 | 9.94E-03 | 1.08E-02 | 1.11E-02 | 1.11E-02 | 1.50E-02 | 3.07E-02 | 6.72E-02 |
| MGEs | transposase | tnpA-01 |  |  |  |  |  |  | 6.16E-04 |
| MGEs | transposase | tnpA-02 |  |  |  |  |  |  | 8.14E-04 |
| MGEs | transposase | tnpA-03 | 1.54E-02 | 2.01E-02 | 2.06E-02 | 2.25E-02 | 2.26E-02 | 1.45E-02 | 2.59E-02 |
| MGEs | transposase | tnpA-04 | 1.46E-04 | 5.37E-04 | 3.38E-04 | 1.04E-04 | 1.60E-02 | 2.63E-02 | 2.67E-02 |
| MGEs | transposase | tnpA-05 | 3.09E-04 | 2.55E-04 | 5.91E-04 | 1.71E-04 | 2.35E-03 | 3.79E-03 | 9.99E-04 |
| MGEs | transposase | IS613 |  |  |  |  |  |  | 1.19E-03 |
| MGEs | transposase | Tp614 |  |  |  |  |  |  | 5.99E-04 |

**Table S4** The shared ARGs among the CK, N, NP, and NPK treatments.

| Gene | CK | N | NP | NPK | Classification | Mechanism |
| --- | --- | --- | --- | --- | --- | --- |
| aacC4 | 4.35E-05 | 2.32E-04 | 1.70E-04 | 1.30E-04 | Aminoglycoside | deactivate |
| aadA | 9.52E-05 | 8.24E-05 | 1.41E-04 | 1.42E-04 | Aminoglycoside | deactivate |
| aadE | 6.65E-05 | 6.27E-05 | 7.67E-05 | 9.92E-05 | Aminoglycoside | deactivate |
| bla1 | 8.54E-04 | 2.97E-04 | 9.72E-04 | 8.31E-04 | Beta_lactamase | deactivate |
| blaCTX-M | 2.07E-04 | 8.53E-04 | 1.02E-03 | 6.34E-04 | Beta_lactamase | deactivate |
| blaSFO | 9.96E-04 | 2.23E-03 | 1.54E-03 | 1.13E-03 | Beta_lactamase | deactivate |
| cphA | 1.64E-02 | 1.57E-02 | 1.32E-02 | 1.13E-02 | Beta_lactamase | deactivate |
| fox5 | 1.72E-03 | 8.57E-04 | 2.68E-03 | 4.69E-03 | Beta_lactamase | deactivate |
| Pbp5 | 3.34E-04 | 2.03E-04 | 2.46E-04 | 1.19E-04 | Beta_lactamase | protection |
| ermB | 1.02E-04 | 1.02E-04 | 8.11E-05 | 5.96E-05 | MLSB | protection |
| lnuA-01 | 2.45E-05 | 4.35E-05 | 3.15E-05 | 4.14E-05 | MLSB | deactivate |
| mphA | 1.57E-03 | 1.73E-03 | 1.15E-03 | 1.79E-03 | MLSB | deactivate |
| msrC-01 | 8.14E-05 | 8.81E-05 | 1.08E-04 | 1.17E-04 | MLSB | efflux |
| oleC | 9.53E-04 | 1.54E-03 | 1.33E-03 | 1.07E-03 | MLSB | efflux |
| vatE | 4.38E-05 | 5.53E-04 | 4.95E-04 | 7.10E-04 | MLSB | deactivate |
| acrA | 5.59E-04 | 4.16E-03 | 4.03E-03 | 3.01E-03 | Multidrug | efflux |
| ceoA | 2.60E-04 | 1.21E-03 | 6.97E-04 | 8.34E-04 | Multidrug | efflux |
| marR-01 | 9.32E-05 | 8.00E-05 | 8.22E-05 | 6.65E-05 | Multidrug | efflux |
| mdtE_yhiU | 8.57E-05 | 1.70E-04 | 9.43E-05 | 1.12E-04 | Multidrug | efflux |
| oprJ | 1.59E-02 | 1.01E-02 | 2.44E-02 | 1.78E-02 | Multidrug | efflux |
| putitive multidrug | 1.94E-03 | 1.66E-03 | 1.45E-03 | 1.43E-03 | Multidrug | efflux |
| qacEdelta1 | 5.21E-05 | 2.61E-04 | 1.43E-04 | 9.65E-05 | Multidrug | efflux |
| tolC | 1.03E-04 | 1.77E-04 | 5.87E-05 | 9.57E-05 | Multidrug | efflux |
| tetM | 7.35E-05 | 4.54E-05 | 6.99E-05 | 2.41E-05 | Tetracycline | protection |
| tetPB | 2.13E-03 | 1.74E-03 | 1.36E-03 | 1.76E-03 | Tetracycline | protection |
| tetS | 7.71E-05 | 1.36E-04 | 9.25E-05 | 6.95E-05 | Tetracycline | protection |
| pncA | 1.31E-03 | 2.51E-03 | 1.15E-03 | 1.08E-03 | Other/Pyrazinamide | unknown |
| sat4 | 4.95E-05 | 8.49E-05 | 4.42E-05 | 3.06E-05 | Other/streptothricin | deactivate |

**Table S5** The shared ARGs among the CK, M, MN, and PM treatments.

| Gene | CK | M | MN | PM | Classification | Mechanism |
| --- | --- | --- | --- | --- | --- | --- |
| aadA | 9.52E-05 | 1.03E-02 | 2.03E-02 | 1.34E-01 | Aminoglycoside | deactivate |
| aadA2 | 9.95E-05 | 8.18E-03 | 2.01E-02 | 1.28E-01 | Aminoglycoside | deactivate |
| aadA9 | 3.37E-05 | 5.83E-04 | 1.39E-03 | 5.72E-03 | Aminoglycoside | deactivate |
| aadE | 6.65E-05 | 1.89E-04 | 8.57E-04 | 2.03E-02 | Aminoglycoside | deactivate |
| blaCTX-M | 2.07E-04 | 2.11E-04 | 4.80E-04 | 8.90E-05 | Beta_lactamase | deactivate |
| cphA | 1.64E-02 | 2.68E-02 | 9.59E-02 | 2.56E-02 | Beta_lactamase | deactivate |
| Pbp5 | 3.34E-04 | 2.03E-04 | 1.77E-04 | 9.95E-05 | Beta_lactamase | protection |
| ermB | 1.02E-04 | 2.54E-04 | 2.01E-04 | 4.95E-02 | MLSB | protection |
| lnuA-01 | 2.45E-05 | 1.83E-04 | 2.47E-04 | 5.36E-03 | MLSB | deactivate |
| lnuB | 3.26E-05 | 5.43E-04 | 8.87E-04 | 1.14E-02 | MLSB | deactivate |
| mphA | 1.57E-03 | 1.72E-03 | 2.66E-03 | 2.43E-03 | MLSB | deactivate |
| oleC | 9.53E-04 | 1.45E-03 | 2.94E-03 | 5.52E-04 | MLSB | efflux |
| pikR2 | 5.31E-04 | 7.82E-04 | 8.93E-04 | 7.34E-04 | MLSB | protection |
| vatE | 4.38E-05 | 4.39E-04 | 1.28E-03 | 5.99E-03 | MLSB | deactivate |
| acrA | 5.59E-04 | 4.89E-04 | 9.18E-04 | 3.24E-03 | Multidrug | efflux |
| qacEdelta1 | 5.21E-05 | 1.26E-02 | 9.12E-03 | 2.06E-01 | Multidrug | efflux |
| ttgA | 7.31E-05 | 1.57E-04 | 1.16E-04 | 5.58E-05 | Multidrug | efflux |
| tetG | 2.96E-04 | 1.55E-02 | 1.73E-02 | 1.04E-01 | Tetracycline | efflux |
| tetM | 7.35E-05 | 1.18E-03 | 2.83E-03 | 5.63E-02 | Tetracycline | protection |
| tetPB | 2.13E-03 | 1.69E-03 | 1.52E-03 | 1.55E-03 | Tetracycline | protection |
| tetS | 7.71E-05 | 6.47E-05 | 7.09E-05 | 1.81E-03 | Tetracycline | protection |
| pncA | 1.31E-03 | 1.73E-03 | 4.70E-03 | 4.28E-03 | Other/Pyrazinamide | unknown |
| sat4 | 4.95E-05 | 1.67E-04 | 2.57E-04 | 7.52E-03 | Other/streptothricin | deactivate |

**Table S6** The fold change of the ARGs in soil samples

| Classification | Mechanism | gene | N | NP | NPK | M | MN |
| --- | --- | --- | --- | --- | --- | --- | --- |
| Aminoglycoside | deactivate | aac(6')-Ib(aka aacA4)-01 |  |  |  | 22.66 | 27.57 |
| Aminoglycoside | deactivate | aac(6')-Ib(aka aacA4)-02 |  |  |  | 31.67 | 14.35 |
| Aminoglycoside | deactivate | aac(6')-Ib(aka aacA4)-03 |  |  |  | 41.51 |  |
| Aminoglycoside | deactivate | aac(6')-II |  |  | 1.19 | 14.27 | 6.03 |
| Aminoglycoside | deactivate | aacA_aphD |  |  |  | 2.51 | 2.07 |
| Aminoglycoside | deactivate | aacC | 38.25 | 47.91 | 50.86 |  | 124.98 |
| Aminoglycoside | deactivate | aacC1 |  |  |  | 8.72 |  |
| Aminoglycoside | deactivate | aacC2 |  |  |  | 5.22 |  |
| Aminoglycoside | deactivate | aacC4 | 6.92 | 4.98 | 3.77 |  | 15.76 |
| Aminoglycoside | deactivate | aadA-01 | 2.15 | 1.68 |  | 57.61 | 102.85 |
| Aminoglycoside | deactivate | aadA-02 |  | 2.36 | 2.08 | 53.43 | 63.41 |
| Aminoglycoside | deactivate | aadA1 |  |  | 1.64 | 93.15 | 124.78 |
| Aminoglycoside | deactivate | aadA-1-01 | 2.07 | 2.99 | 2.77 | 8.20 | 7.26 |
| Aminoglycoside | deactivate | aadA-1-02 | 3.40 |  |  | 10.90 | 12.90 |
| Aminoglycoside | deactivate | aadA2-01 | 6.17 |  |  | 111.48 | 207.01 |
| Aminoglycoside | deactivate | aadA2-02 |  | 2.75 |  | 22.24 | 152.82 |
| Aminoglycoside | deactivate | aadA2-03 |  | 8.37 |  | 270.39 | 421.30 |
| Aminoglycoside | deactivate | aadA5-01 |  |  |  | 17.27 | 20.09 |
| Aminoglycoside | deactivate | aadA5-02 | 3.13 | 3.05 |  | 13.07 |  |
| Aminoglycoside | deactivate | aadA9-01 |  | 1.30 |  | 15.55 | 65.81 |
| Aminoglycoside | deactivate | aadA9-02 |  |  |  | 8.45 |  |
| Aminoglycoside | deactivate | aadD |  |  |  |  | 3.49 |
| Aminoglycoside | deactivate | aadE | 0.99 | 1.17 | 1.55 | 2.88 | 13.00 |
| Aminoglycoside | deactivate | aphA1(aka kanR) |  |  |  | 18.41 | 27.41 |
| Aminoglycoside | deactivate | spcN-02 |  |  | 1.25 |  | 4.46 |
| Aminoglycoside | deactivate | str |  |  |  | 3.64 | 6.24 |
| Aminoglycoside | deactivate | strB | 8.33 |  |  | 107.66 | 156.18 |
| Beta_lactamase | deactivate | ampC-01 |  | 2.74 | 2.09 |  |  |
| Beta_lactamase | deactivate | ampC-02 | 2.76 |  |  |  | 3.91 |
| Beta_lactamase | deactivate | ampC-04 | 27.62 | 20.62 | 21.15 |  |  |
| Beta_lactamase | deactivate | ampC-06 |  |  |  |  | 2.24 |
| Beta_lactamase | deactivate | bla1 | 0.31 | 1.13 | 0.97 | 0.87 | 0.69 |
| Beta_lactamase | deactivate | blaCMY2-02 |  | 6.38 | 7.35 |  | 11.47 |
| Beta_lactamase | deactivate | blaCTX-M-01 |  | 4.94 | 8.24 |  | 17.35 |
| Beta_lactamase | deactivate | blaCTX-M-02 | 4.15 | 3.14 | 3.98 |  | 10.25 |
| Beta_lactamase | deactivate | blaCTX-M-04 | 7.88 | 6.04 | 7.16 | 2.08 |  |
| Beta_lactamase | deactivate | blaCTX-M-05 |  | 6.43 |  |  |  |
| Beta_lactamase | deactivate | bla-L1 |  |  |  |  | 3.13 |
| Beta_lactamase | deactivate | blaOCH |  |  |  |  | 3.56 |
| Beta_lactamase | deactivate | blaOXA1_blaOXA30 |  |  |  |  | 4.57 |
| Beta_lactamase | deactivate | blaOXA10-02 |  |  |  | 4.54 | 4.85 |
| Beta_lactamase | deactivate | blaOXY | 6.86 | 4.42 | 4.22 |  | 26.32 |
| Beta_lactamase | deactivate | blaPER | 2.63 | 2.47 | 2.37 |  | 2.30 |
| Beta_lactamase | deactivate | blaPSE |  |  |  | 3.67 | 5.09 |
| Beta_lactamase | deactivate | blaSFO | 2.25 | 1.52 | 1.12 | 1.18 |  |
| Beta_lactamase | deactivate | blaSHV-01 | 1.99 | 1.55 | 1.00 |  |  |
| Beta_lactamase | deactivate | blaTEM | 8.03 |  | 4.97 | 3.93 |  |
| Beta_lactamase | deactivate | blaVIM | 2.64 |  | 1.80 |  | 3.10 |
| Beta_lactamase | deactivate | cphA-01 | 0.94 | 0.79 | 0.68 | 1.61 | 5.63 |
| Beta_lactamase | deactivate | cphA-02 | 4.48 | 2.35 | 0.37 | 2.28 | 6.57 |
| Beta_lactamase | deactivate | fox5 |  | 1.74 | 3.03 | 2.13 |  |
| Beta_lactamase | protection | Pbp5 | 0.66 | 0.79 | 0.38 | 0.67 | 0.45 |
| Beta_lactamase | protection | penA | 2.60 | 4.54 | 6.24 |  |  |
| FCA | deactivate | catB3 |  |  |  | 17.42 | 16.63 |
| FCA | efflux | cmlA1-01 | 6.64 |  |  |  |  |
| FCA | efflux | cmlA1-02 | 19.97 | 9.29 |  |  |  |
| FCA | efflux | cmx(A) |  |  |  |  | 18.16 |
| FCA | efflux | floR | 26.18 |  |  | 94.55 |  |
| MLSB | deactivate | ereA |  |  |  | 2.25 | 9.07 |
| MLSB | protection | erm(34) |  | 1.75 |  |  |  |
| MLSB | protection | erm(35) |  |  |  |  | 3.72 |
| MLSB | protection | ermA |  |  |  |  | 3.10 |
| MLSB | protection | ermB | 0.98 | 0.71 | 0.52 | 1.73 | 1.96 |
| MLSB | protection | ermC |  |  |  |  | 3.67 |
| MLSB | protection | ermF |  |  |  | 41.97 | 53.96 |
| MLSB | protection | ermK-01 |  |  |  |  | 3.07 |
| MLSB | protection | ermT-01 |  |  |  |  | 4.27 |
| MLSB | protection | ermT-02 |  |  |  | 2.35 | 5.58 |
| MLSB | protection | ermX |  | 2.65 |  | 7.91 |  |
| MLSB | deactivate | lnuA-01 | 1.77 | 1.06 | 1.68 | 6.35 | 9.86 |
| MLSB | deactivate | lnuB-01 |  |  |  | 11.02 | 18.14 |
| MLSB | deactivate | lnuB-02 |  | 1.50 |  | 11.21 | 15.28 |
| MLSB | efflux | matA_mel |  |  |  | 6.77 |  |
| MLSB | efflux | mefA |  |  |  | 3.41 | 13.45 |
| MLSB | deactivate | mphA-01 | 1.43 | 0.92 | 1.05 | 1.26 | 1.67 |
| MLSB | deactivate | mphA-02 |  |  | 1.47 |  | 3.49 |
| MLSB | efflux | msrC-01 | 1.07 | 1.33 | 1.32 | 0.96 | 0.74 |
| MLSB | efflux | oleC | 5.60 | 4.97 | 4.06 | 5.37 | 11.25 |
| MLSB | protection | pikR1 |  |  |  |  | 1.24 |
| MLSB | protection | pikR2 | 1.74 |  |  | 1.52 | 1.74 |
| MLSB | deactivate | vatE-01 | 11.75 | 8.89 | 17.44 | 4.73 | 29.40 |
| MLSB | deactivate | vatE-02 |  |  |  |  | 3.29 |
| MLSB | deactivate | vgb-01 |  |  |  |  | 2.01 |
| Multidrug | efflux | acrA-01 |  | 3.44 |  |  |  |
| Multidrug | efflux | acrA-02 |  |  |  |  | 3.32 |
| Multidrug | efflux | acrA-04 | 4.33 | 3.98 | 4.23 | 0.87 |  |
| Multidrug | efflux | acrA-05 | 73.19 | 85.56 | 111.28 |  | 65.38 |
| Multidrug | efflux | ceoA | 4.73 | 2.86 | 3.54 | 0.78 |  |
| Multidrug | efflux | cmr |  |  | 28.33 |  |  |
| Multidrug | efflux | emrD |  |  | 26.39 |  |  |
| Multidrug | efflux | marR-01 | 0.94 | 1.04 | 0.76 | 1.03 |  |
| Multidrug | efflux | mdtE_yhiU | 2.08 | 1.11 | 1.35 |  | 4.15 |
| Multidrug | efflux | mepA | 48.87 | 30.22 | 30.27 | 11.04 | 49.96 |
| Multidrug | efflux | mexA |  |  | 0.99 |  | 7.39 |
| Multidrug | efflux | mexE | 10.09 | 6.63 | 8.00 |  | 17.81 |
| Multidrug | efflux | mtrC-01 |  |  | 7.87 |  | 2.97 |
| Multidrug | efflux | mtrC-02 |  |  | 2.08 |  |  |
| Multidrug | efflux | mtrD-03 | 15.52 | 7.38 | 6.64 |  | 10.52 |
| Multidrug | efflux | oprD | 106.12 | 78.90 | 84.09 | 56.57 | 111.29 |
| Multidrug | efflux | oprJ | 0.64 | 1.51 | 1.11 | 1.10 |  |
| Multidrug | efflux | putitive multidrug | 0.85 | 0.74 | 0.72 | 0.80 | 0.54 |
| Multidrug | efflux | qacEdelta1-01 | 5.22 | 2.75 | 1.91 | 124.62 | 147.70 |
| Multidrug | efflux | qacEdelta1-02 |  |  |  | 327.27 |  |
| Multidrug | efflux | qacH-01 | 4.52 |  |  |  | 12.33 |
| Multidrug | efflux | qacH-02 |  |  |  | 2.12 |  |
| Multidrug | efflux | rarD-02 |  |  | 1.17 |  |  |
| Multidrug | efflux | tolC-02 | 5.57 |  | 4.83 |  | 20.81 |
| Multidrug | efflux | tolC-03 | 1.91 | 1.00 |  |  |  |
| Multidrug | efflux | ttgA |  | 1.39 |  | 2.17 | 1.42 |
| Multidrug | efflux | ttgB |  | 11.11 |  | 7.27 |  |
| Multidrug | efflux | yidY_mdtL-01 |  |  | 2.64 |  |  |
| Sulfonamide | deactivate | dfrA1 |  |  |  | 15.49 | 24.46 |
| Sulfonamide | protection | sul1 |  |  |  |  | 4.30 |
| Sulfonamide | protection | sul2 |  | 5.74 | 4.02 | 216.99 | 303.77 |
| Sulfonamide | protection | sulA-folP-01 |  |  |  |  | 4.14 |
| Sulfonamide | protection | sulA-folP-03 | 3.12 | 4.46 |  |  | 8.37 |
| Tetracycline | protection | tet(32) |  |  |  | 12.29 | 10.66 |
| Tetracycline | unknown | tet(34) |  |  | 1.43 |  | 2.82 |
| Tetracycline | protection | tet(36)-01 |  |  |  |  | 4.23 |
| Tetracycline | protection | tet(36)-02 |  |  |  |  | 4.26 |
| Tetracycline | efflux | tetA-02 | 2.95 | 2.07 |  |  | 37.75 |
| Tetracycline | efflux | tetB-02 | 4.27 | 3.55 | 10.18 | 7.38 | 41.90 |
| Tetracycline | efflux | tetD-01 | 2.98 |  |  |  |  |
| Tetracycline | efflux | tetD-02 |  | 5.69 | 4.43 |  | 4.76 |
| Tetracycline | efflux | tetG-01 | 1.17 |  | 1.31 |  |  |
| Tetracycline | efflux | tetG-02 | 3.76 |  | 2.17 | 229.16 | 259.11 |
| Tetracycline | efflux | tetL-02 |  |  |  | 8.55 |  |
| Tetracycline | protection | tetM-01 | 0.63 | 0.97 |  | 12.19 | 24.06 |
| Tetracycline | protection | tetM-02 |  |  | 1.20 | 14.96 | 41.63 |
| Tetracycline | protection | tetO-01 | 1.79 | 1.33 |  |  | 17.10 |
| Tetracycline | efflux | tetPA |  |  |  | 7.22 | 15.06 |
| Tetracycline | protection | tetPB-01 | 0.81 | 0.64 | 1.01 | 1.02 |  |
| Tetracycline | protection | tetPB-02 |  |  |  |  | 9.61 |
| Tetracycline | protection | tetPB-03 |  |  |  |  | 20.97 |
| Tetracycline | protection | tetPB-04 | 0.95 | 1.04 | 1.42 | 0.82 | 0.74 |
| Tetracycline | protection | tetPB-05 | 0.71 | 0.59 | 0.69 | 0.65 | 0.40 |
| Tetracycline | protection | tetQ |  |  |  | 10.28 | 8.33 |
| Tetracycline | efflux | tetR-02 |  |  | 1.00 |  | 23.30 |
| Tetracycline | protection | tetS | 1.84 | 1.21 | 0.87 | 0.81 | 0.95 |
| Tetracycline | protection | tetT | 1.38 |  |  | 7.78 | 19.44 |
| Tetracycline | unknown | tetX | 9.52 |  |  | 84.43 | 123.84 |
| Vancomycin | protection | vanB-01 | 26.86 | 14.46 | 19.40 |  | 26.35 |
| Vancomycin | protection | vanC-01 | 3.58 |  |  |  |  |
| Vancomycin | protection | vanC-03 | 57.41 |  | 46.50 | 50.78 | 98.57 |
| Vancomycin | protection | vanHB |  | 9.61 | 14.24 | 10.95 | 24.93 |
| Vancomycin | protection | vanRA-01 |  | 1.70 | 1.07 | 1.42 | 1.13 |
| Vancomycin | protection | vanSB |  | 2.88 | 3.80 | 2.64 | 5.51 |
| Vancomycin | protection | vanTC-02 |  | 3.12 | 6.35 |  |  |
| Vancomycin | protection | vanYD-01 |  |  |  |  | 5.53 |
| other/bacitracin | deactivate | bacA-01 |  |  |  |  | 4.57 |
| other/Pyrazinamide | unknown | pncA | 1.87 | 0.88 | 0.84 | 1.34 | 3.30 |
| other/streptothricin | deactivate | sat4 | 2.48 | 1.26 | 0.89 | 4.45 | 7.53 |
